# HB-EGF enhances collective cell migration via spatial coordination of traction

**DOI:** 10.1063/5.0326208

**Published:** 2026-06-23

**Authors:** Jonah J. Spencer, Emily Rhine, Pamela K. Kreeger, Jacob Notbohm

**Affiliations:** 1 Biophysics Program, University of Wisconsin–Madison, Madison, Wisconsin 53706, USA; 2 Department of Mechanical Engineering, University of Wisconsin–Madison, Madison, Wisconsin 53706, USA; 3 Department of Biomedical Engineering, University of Wisconsin–Madison, Madison, Wisconsin 53706, USA; 4 Department of Pathology and Laboratory Medicine, University of Wisconsin School of Medicine and Public Health, Madison, Wisconsin 53705, USA; 5 University of Wisconsin Carbone Cancer Center, University of Wisconsin School of Medicine and Public Health, Madison, Wisconsin 53705, USA

## Abstract

An important process in wound healing is re-epithelialization, wherein cells collectively migrate to cover the wounded area. Here, we investigate how cellular forces lead to the migration of an epithelial monolayer in a wound healing assay. We report that heparin-bound epidermal growth factor (HB-EGF) increased the rate of collective migration in a phospholipase C-dependent manner through a combination of increased cell speed and straighter, more coordinated motion. Using traction force microscopy, we found that HB-EGF increased the forces within the cell monolayer, producing a competition between elevated traction at the edge of the monolayer and elevated stress within the bulk of the monolayer. To investigate how this interplay led to faster monolayer migration, we used a theoretical model for collective cell migration, which, when compared against the experimental data, suggested faster migration resulted from increased active propulsive forces at both the leading edge and within the bulk of the cell monolayer. Experimental analysis of actin stress fibers and vinculin foci supported inferences made from the model. Combined, our results support that HB-EGF induced a greater magnitude of traction for cells at the edge of the monolayer and aligned the direction of traction for cells within the bulk, thereby leading to faster and more persistent collective migration.

## INTRODUCTION

Cell migration is a fundamental component of development, wound healing, and disease processes such as metastasis. While many studies have focused on the biological factors that direct migration, it is inherently a physical process, meaning that migration ultimately results from cell forces. Some processes such as re-epithelialization result when multiple cells maintain cell–cell contact and move as a collective unit.^1–4^ Within a cellular collective, forces produced by each cell are transmitted both to the substrate and to the neighboring cells, resulting in a complex and poorly understood relationship between force and motion. Challenges include that there are non-local couplings between force and motion, multiple simultaneous force-generating mechanisms, nonlinear mechanical feedback loops, and cellular activity that varies over time and space.^5–11^ As a result, it is difficult to interpret experimental data for cell forces, especially to infer how the forces produce collective motion. Due to the inability to accurately predict how cell-generated forces cause motion, it is not yet possible to predict how specific perturbations can alter processes like wound closure.

To construct a clearer representation of the relation between forces and velocity, detailed analysis of cellular force production and transmission is needed. The forces vary with position in the cell collective. In general, the tractions applied by the cells to the substrate fluctuate strongly over space.^12^ At the edge of a cell monolayer, the traction often has a greater magnitude compared to within the bulk, and tractions at the edge commonly pull the cells into the free space.^11–14^ Given that the tractions are in mechanical equilibrium with the intercellular stresses within the monolayer,^15^ the propulsion caused by traction at the edge of the monolayer is resisted by intercellular tensile stress within the bulk.^12,15^ Additionally, cells tend to coordinate so as to align the direction of traction with that of their neighbors,^16^ and tractions are often elevated near cellular protrusions at the edge of the monolayer.^17^ While these observations describe common trends and help to establish a qualitative picture of how cellular forces vary within the monolayer, quantitative understanding is still lacking. A major challenge is that the forces are a combination of active (i.e., resulting from actomyosin contraction within each cell) and passive (i.e., resulting from viscoelasticity associated with cellular flows and deformations).^18–20^ Whereas the cell–substrate tractions can be measured, the experiments measure the total traction, which is a sum of active and passive. In general, it is not yet possible to decouple active from passive forces, meaning it is not yet possible to relate cell forces to velocity quantitatively. Hence, it still remains unclear how cells coordinate over space to produce spatial patterns in force and, in turn, how those forces lead to collective migration.

In order to develop such an understanding, we consider the process of re-epithelialization, wherein keratinocytes move as a collective to cover a dermal wound.^1–4^ This biological setting has several advantages, including that it is essentially a two-dimensional process and has been well characterized with respect to factors that result in different degrees of collective motion. For example, we and others^21–27^ have shown that epidermal growth factor (EGF) stimulates collective migration and wound closure, with the rate of collective migration dependent on EGF dose and substrate stiffness.^23^ Our analysis determined that displacement of the leading edge depended more on individual cell persistence than cell speed, but in our prior work, we did not examine forces. We determined that persistence can be increased by immobilizing EGF to the polystyrene culture substrate, and this increase in persistence results from phospholipase C (PLC) activation.^24^ Here, we opted for a simpler method to activate PLC by treating cells with soluble heparin-bound EGF (HB-EGF).^28^

In this study, we first tested the effect of HB-EGF on collective migration, and we verified that it led to an increase in the rate and persistence of migration in a PLC-dependent manner. Next, we experimentally measured the traction at the cell-to-substrate interface. Because the measured traction represents an inseparable combination of active and passive components, we interpreted the observations through a theoretical model. From the theory, we predicted that HB-EGF led to changes in both the magnitude and spatial distribution of active tractions produced by the cells. Finally, we tested this prediction by examining the actin cytoskeleton and focal adhesions.

## RESULTS

### Effect of HB-EGF on cell velocity in an expanding monolayer

**FIG. 1.**
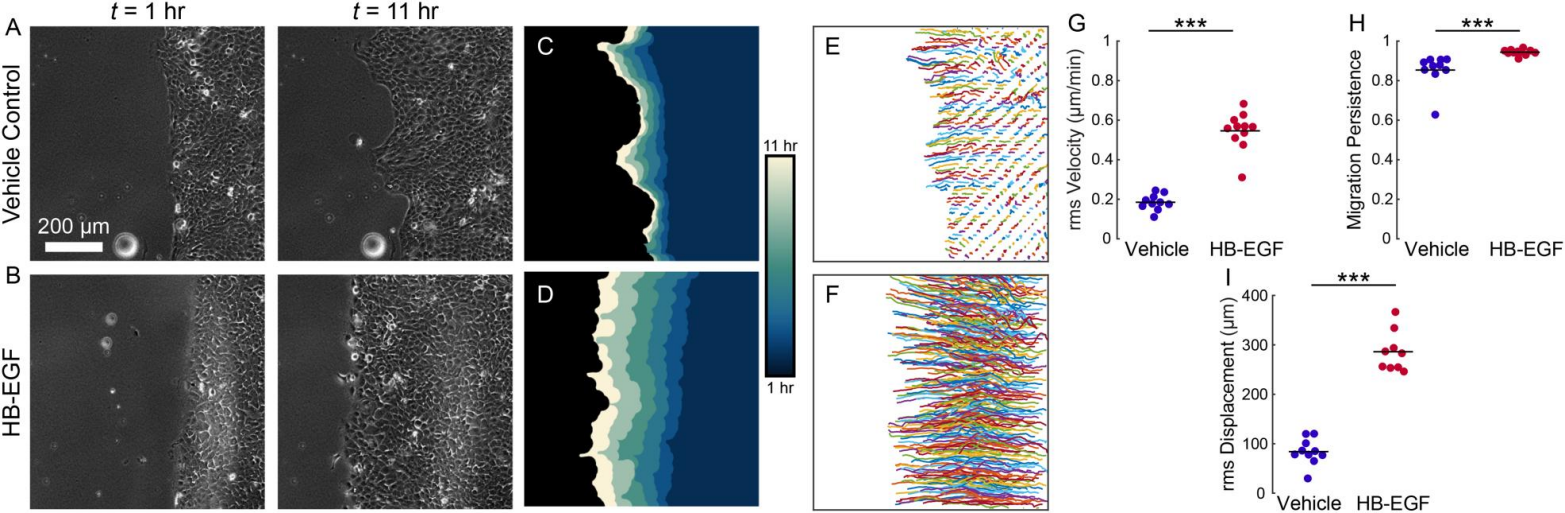
Collective migration in response to HB-EGF in expanding cell monolayers. (a) and (b) Phase contrast images of representative monolayers treated with vehicle control and HB-EGF. Monolayers are shown at t=1 hr and t=11 hr, demonstrating greater migration in the HB-EGF-treated monolayer. (c) and (d) Segmentation of the representative expanding monolayers at times ranging from 1 to 11 hr post-treatment, for vehicle control and HB-EGF. Representative cell trajectories of expanding monolayers treated with (e) vehicle or (f) HB-EGF. Trajectories correspond to times 1–11 hr post-treatment. Scatter plots of (g) rms velocity ( $p=1.8\times 10^{-4}$), (h) migration persistence ( $p=1.8\times 10^{-4}$), and (i) rms cell displacement ( $p=2.2\times 10^{-5}$). In all scatter plots, each point is an average over an independent field of view; the black bar represents the mean over all fields of view.

Our experiments used a wound closure assay wherein cells were seeded on compliant substrates against barriers and grown to confluence, at which time the barriers were removed, allowing the cells to migrate collectively to fill the free space (Fig. 6, Appendix C). To begin, we examined the effect of HB-EGF to confirm that migration was stimulated in a manner similar to prior studies using immobilized EGF.^22^ Upon barrier removal, the cells were treated with either vehicle control or HB-EGF and imaged by time-lapse phase contrast microscopy as they migrated into the free space [Figs. 1(a) and 1(b)]. The point at which the barrier was removed and the treatments were added was defined as t=0 hr. To visualize the rate of collective migration, the edge of the cell monolayer was identified at different points in time by segmentation using images of cell nuclei (described in the section on Methods). Results showed that the cells treated with HB-EGF had greater overall leading edge displacement [Figs. 1(c) and 1(d)]. Cell velocities were also quantified by applying image correlation to the phase contrast images. Representative cell trajectories, computed from the velocity data, showed faster migration in cells treated with HB-EGF [Figs. 1(e) and 1(f)].

To quantify these observations, we computed the root mean square (rms) of each cell's velocity and took the average over each field of view. In vehicle control conditions, the rms velocity was approximately 0.2 *μ*m/min, whereas under HB-EGF treatment, the rms velocity was nearly 0.6 *μ*m/min [Fig. 1(g)]. Inspection of the trajectories in Figs. 1(e) and 1(f) showed that in addition to having longer trajectories, cells treated with HB-EGF also had highly straight trajectories. This observation is consistent with prior studies that showed that migration persistence (defined as the Euclidean displacement divided by total distance traveled for each cell) is important for fast wound closure.^23–25^ In our experiments, the persistence was relatively high in vehicle control conditions, with an average value of ≈0.82. In response to HB-EGF, persistence was significantly higher, with an average value of ≈0.93, indicating increased intercellular coordination of velocity [Fig. 1(h)]. To quantify the combined effects of elevated speed and persistence caused by HB-EGF, we computed the displacement of each cell over 10 hr of migration and computed the rms of all displacements in each field of view. In vehicle control conditions, cells traveled ≈85 *μ*m, whereas cells treated with HB-EGF traveled ≈290 *μ*m [Fig. 1(i)]. This ≈3.4× increase in displacement is greater than the fractional increases in rms velocity ( ≈2.7×), suggesting that the greater rate of monolayer expansion resulted from a combination of increased speed and increased persistence upon treatment with HB-EGF. Additionally, we compared the effects of soluble HB-EGF to immobilized EGF, which was identified previously to lead to faster and straighter collective migration.^24^ Results showed that the migration was similar in response to both immobilized EGF and soluble HB-EGF, namely, the rms velocity, migration persistence, and rms displacement of the edge of the monolayer were all larger compared to the vehicle control for both immobilized EGF and HB-EGF (Fig. 7, Appendix C). Given this similarity and because HB-EGF does not require a time-consuming immobilization procedure, we chose to focus on soluble HB-EGF for the remainder of our study.

### Effect of PLC inhibition on HB-EGF-stimulated collective migration

**FIG. 2.**
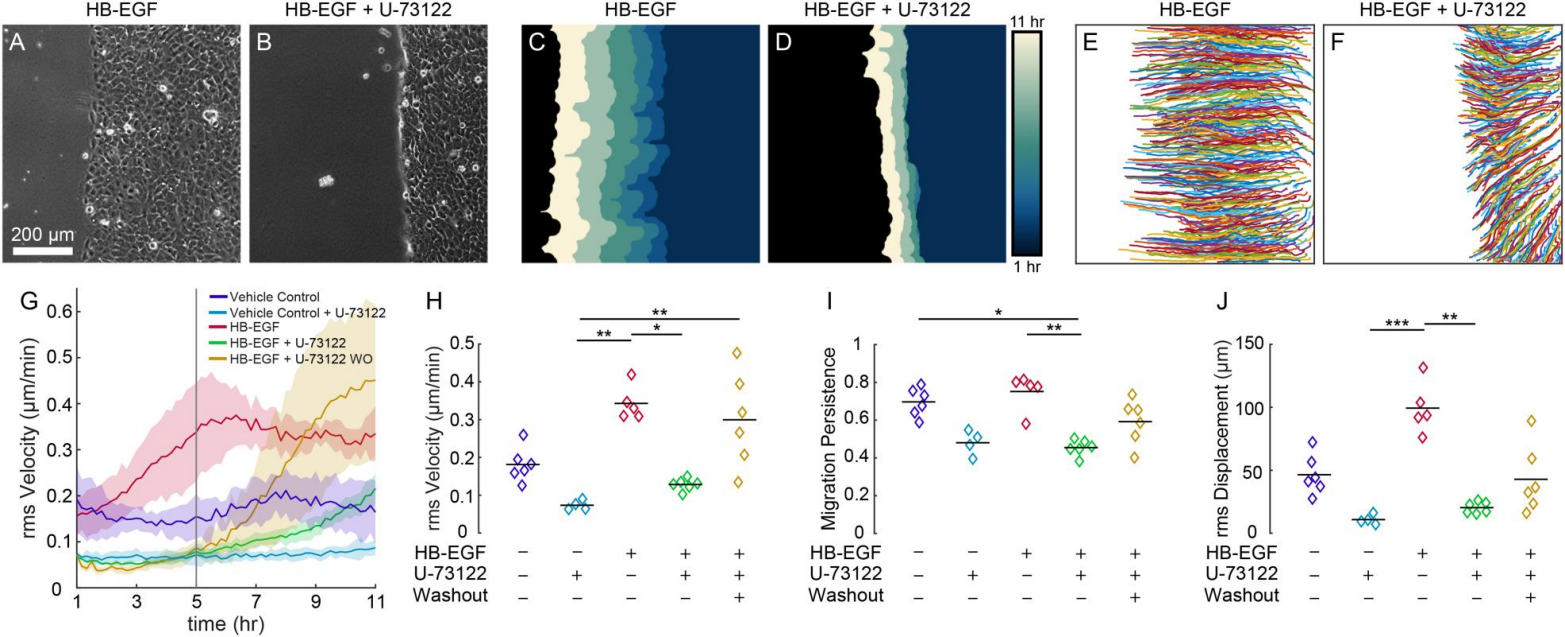
Effect of PLC inhibition on collective migration in expanding cell monolayers. (a) and (b) Phase contrast images demonstrating cell morphology in expanding monolayers treated with HB-EGF or HB-EGF and U-73122 ( t=7 hr). (c) and (d) Segmentation of representative expanding monolayer at times ranging from 1 to 11 hr post-treatment for HB-EGF or HB-EGF and U-73122. (e) and (f) Representative cell trajectories of expanding monolayers treated with HB-EGF or HB-EGF and U-73122. Trajectories correspond to times 1–11 hr post-treatment. (g) Root mean square velocity over time for vehicle control, vehicle control + U-73122, HB-EGF, HB-EGF + U-73122, and HB-EGF + U-73122 washout. Lines are means across different fields of view, and shading represents standard deviation. The vertical line at t=5 hr indicates the time of the washout for the data labeled HB-EGF + U-73122 WO. Scatter plots of (h) rms velocity, (i) migration persistence, and (j) rms cell displacement of cell monolayers treated with vehicle control, vehicle control + U-73122, HB-EGF, HB-EGF + U-73122, and HB-EGF + U-73122 washout. In all scatter plots, dots indicate data from an independent field of view averaged over t=5–11 hr post-treatment and barrier removal. Black bars represent means over multiple fields of view. For the washout in (g)–(j), at t=5 hr HB-EGF + U-73122, the medium was changed to medium containing only HB-EGF. Statistical results of the data in panels (h)–(j) are located in Appendix D.

Given our prior studies with immobilized EGF^24,25^ suggested that PLC activation stimulated highly persistent migration and HB-EGF is known to stimulate PLC,^28^ we examined if the robust migration in our studies was PLC-dependent. Cells were seeded against barriers and treated with either HB-EGF or HB-EGF with U-73122, which inhibits receptor-mediated activation of PLC. After removal of the barrier, the cells were imaged as they migrated into free space [Figs. 2(a) and 2(b)]. As above, time t=0 is the time of both the treatments and barrier removal. Segmentation of representative cell monolayers at different time points demonstrated a hindered migration rate, with cells treated with U-73122 showing a drastic reduction in migration [Figs. 2(c) and 2(d)]. Further visualization of decreased migration is apparent in representative cell trajectories over 10 hr of migration [Figs. 2(e) and 2(f)]. In addition to visibly less migration, representative trajectories of the PLC-inhibited monolayer appeared less aligned outwards into the free space. To quantify these observations, we computed rms velocity, migration persistence, and rms displacement as above, with results showing a statistically significant decrease in all three for cell monolayers treated with HB-EGF + U-73122 compared to monolayers treated with only HB-EGF [Figs. 2(g)–2(j)].

A control was performed by treating with U-73122 without HB-EGF. While rms velocity, migration persistence, and rms displacement appeared to trend lower for U-73122 compared to vehicle control, the apparent differences were not statistically significant [Figs. 2(g)–2(j)]. The apparent decrease could be related to a small degree of PLC activation resulting from our use of 1% serum, while the prior studies with immobilized EGF were conducted in serum-free media.^24^ To demonstrate reversibility, an HB-EGF + U-73122 washout (WO) experiment was performed. In the washout experiment, the cells initially experienced the same conditions as HB-EGF + U-73122. At t=5 hr [signified in Fig. 2(g) by the vertical line], medium containing HB-EGF and U-73122 was replaced with medium containing only HB-EGF. Upon washing out the U-73122 inhibitor, the monolayer recovered its velocity [Fig. 2(g)]. For further quantification, data were analyzed over t=5–11 hr (data from t=1–5 hr are reported in Fig. 8, Appendix C). For rms velocity [Fig. 2(h)], migration persistence [Fig. 2(i)], and rms displacement [Fig. 2(j)], data for HB-EGF + U-73122 WO were not statistically different compared to data from cells treated with HB-EGF. These data support prior findings with immobilized EGF^24,25^ that the robust migration in response to HB-EGF is PLC-dependent.

### Distribution of cell–substrate traction over distance from the edge of the monolayer

**FIG. 3.**
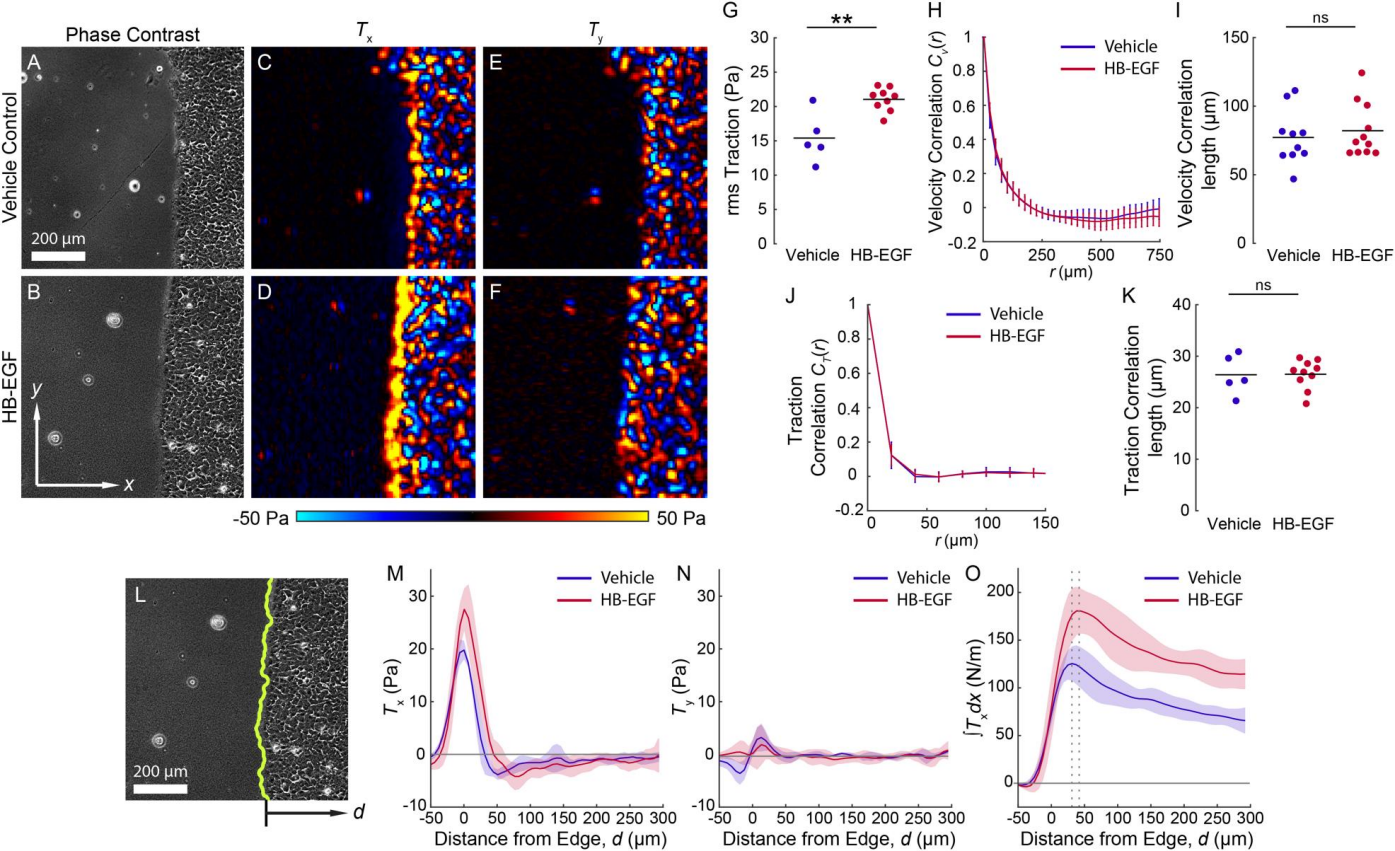
Spatial distributions of velocity and traction in expanding cell monolayers. (a) and (b) Phase contrast images of expanding HaCaT monolayers treated with vehicle control and HB-EGF ( t=1 hr). (c) and (d) Color maps of traction in the *x* direction for vehicle control and HB-EGF. (e) and (f) Color maps of traction in the *y* direction for vehicle control and HB-EGF. (g) Scatter plot of rms traction ( $p=7.0\times 10^{-3}$). (h) Spatial correlation of velocity; error bars represent standard deviations. (i) Scatter plot of velocity correlation length ( p=0.73). (j) Spatial correlation of single-cell traction; error bars represent standard deviations. (k) Scatter plot of traction correlation length ( p=0.95). (l) Phase contrast image of an HB-EGF-treated expanding monolayer with a yellow line indicating the edge of the monolayer, which defines the location d=0, where *d* is the distance from the edge of the cell monolayer ( t=1 hr). Plots of the *x* component of traction (m), the *y* component of traction (n), and integral of $T_{x}$ (o) as a function of distance from the edge of an expanding monolayer. Lines represent means over multiple independent fields of view (vehicle control n=5, HB-EGF n=10) and shading represents standard deviation. In the scatter plots, each point is an average over an independent field of view; black bars represent means. Color maps show tractions at t=1 hr, all other plots are averages over t=1–11 hr for each position.

Following our observations showing that both migration speed and persistence affect the rate of collective migration, we next aimed to identify the forces responsible. To this end, we used traction force microscopy to quantify the cell–substrate tractions during collective migration. We visualized the traction components in the *x* and *y* directions, which were defined as perpendicular and parallel to the wound edge, respectively. Visual examination showed that cells treated with HB-EGF had slightly brighter maps of traction than vehicle control, especially in the *x* direction [Figs. 3(a)–3(f)], which is reasonable given the observation that HB-EGF caused faster migration. To quantify this observation, we computed the rms of traction. Cell monolayers treated with HB-EGF produced greater rms traction ( ≈22 Pa) than vehicle control [ ≈15 Pa, Fig. 3(g)].

To investigate further, we considered the observation that the HB-EGF treatment led to straighter, more persistent migration, indicating enhanced intercellular coordination. How cells coordinate their force generation and transmission between neighbors is still an area of active exploration, but prior studies identified that one cause of intercellular coordination is that cells tend to align the direction of traction that each cell applies to the substrate.^16,29^ Given this prior finding, we hypothesized that in expanding monolayers, HB-EGF would lead to an increased intercellular coordination of both velocity and traction. To quantify the coordination, we measured the spatial correlation lengths of velocity and traction. Surprisingly, the results showed no difference between vehicle control and HB-EGF for either correlation length [Figs. 3(h)–3(k)]. To investigate further, we also performed one experiment with confined 1 mm cell island geometries, wherein results were largely consistent with findings reported previously. In particular, HB-EGF led to elevated correlation lengths of both velocity and traction (Appendix A and Fig. 9 of Appendix C). Hence, the effects of HB-EGF may depend on the boundary conditions of the monolayer, and the effect of HB-EGF on the cellular forces in an unconfined setting is not intercellular coordination of tractions, as was identified in a prior study with confined boundaries.^16^ This suggests that for our study of expanding cell monolayers, a different analysis of the cellular forces will be needed.

We turned back to the color maps of traction [Figs. 3(a)–3(f)] and observed that HB-EGF appeared to increase primarily the *x* component of traction ( $T_{x}$) and that the most elevated values of $T_{x}$ were near the edge of the cell monolayer. To quantify this observation, we used segmentation to identify the edge of the cell monolayer and defined *d* to be the distance along the *x* direction from the edge of the monolayer [Fig. 3(l)]. The resulting dataset was a series of curves of traction ( $T_{x}$ and $T_{y}$) over distance *d*, with different curves corresponding to different *y* positions. The curves for $T_{x}$ and $T_{y}$ were averaged over *y* position, resulting in curves quantifying the average of $T_{x}$ and $T_{y}$ against distance *d*. This spatial averaging potentially smooths over information at the length scale of each cell, but it is an acceptable choice given that our focus is on the migration of the entire collective. Due to the methodology of segmentation, in which cell nuclei were used to define the edge of the cell layer, some cells at the edge had large lamellipodia, causing them to apply tractions beyond the segmented edge. To account for tractions applied beyond the boundary by cell lamellipodia, the data were plotted beginning at d=−50 *μ*m. The sign convention used for traction is the traction applied by the cells to the substrate, meaning positive $T_{x}$ indicates cells propelling themselves in the negative *x* direction, i.e., into the free space. The plot of $T_{x}$ matched the visual observations from Figs. 3(g) and 3(h), namely, that near the edge (i.e., −50<d<50 *μ*m), $T_{x}$ was greater for HB-EGF cell monolayers compared to vehicle control [Fig. 3(m)]. For d>50 *μ*m, $T_{x}$ was slightly negative for both vehicle control and HB-EGF, meaning that for d>50 *μ*m the traction acted like a friction that resisted the collective migration. The plots of $T_{y}$ showed a slight fluctuation at the edge ( −50<d<50 *μ*m), but the data were not notably different from zero for both HB-EGF and vehicle control conditions [Fig. 3(n)].

We also considered that tensile stresses build up within the cell layer,^12,15^ which could potentially impede monolayer expansion. To quantify the stresses, we computed the integral of $T_{x}$ over distance, as in prior work.^12^ For both vehicle control and HB-EGF-treated cell monolayers, the integral of traction increased with distance *d*, indicating a buildup of tensile stress [Fig. 3(o)], which is common in cell monolayers.^11,12^ Interestingly, the buildup of stress (indicated by the magnitude of $\int T_{x}dx$) was greater for cell monolayers treated with HB-EGF compared to vehicle control [Fig. 3(o)]. Finally, we repeated these analyses in PLC-inhibited monolayers, with results showing that the inhibitor largely showed trends that were similar to vehicle control conditions at early time points (Fig. 10, Appendix C). In summary, HB-EGF led to elevated traction at the leading edge of the monolayer and elevated tensile stress within the monolayer.

### Velocity, traction, and stress predicted by a theoretical model for a collectively migrating cell monolayer

From the experimental data alone, it is unclear exactly how the HB-EGF caused faster migration. The greater traction at the edge caused by HB-EGF [Fig. 3(m)] would cause faster collective migration, but the greater tensile stress within the bulk would cause slower migration. More generally, traction and stress must balance due to the principle of equilibrium, meaning there is always an interplay between traction and stress. To study this interplay and to decipher how HB-EGF caused faster collective migration, we turned to a theoretical model.

**FIG. 4.**
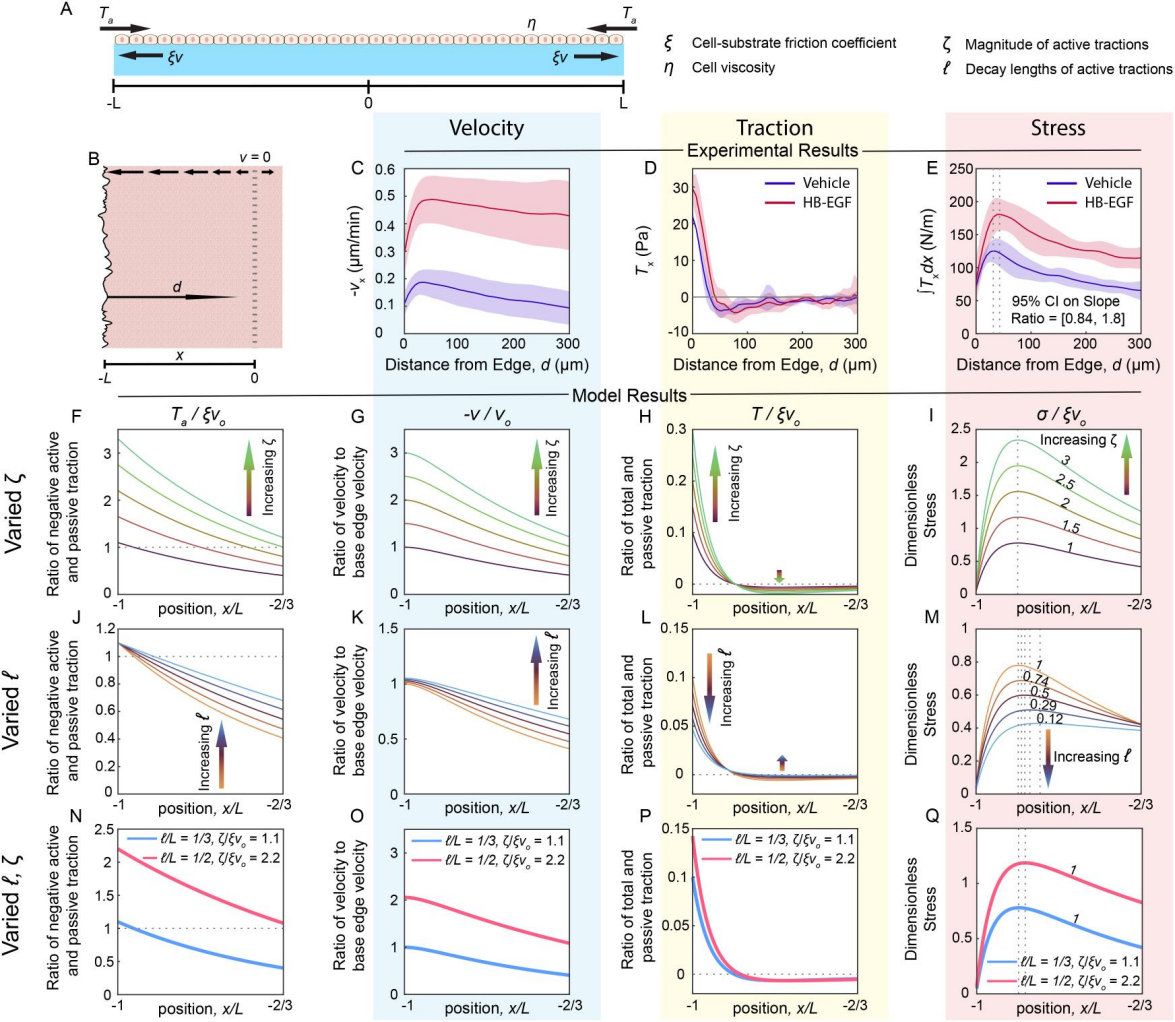
Theoretical model relating forces and velocities in the expanding cell monolayer. (a) Schematic of cellular forces and variables considered in the model. Arrows represent the forces applied by the cell layer onto the substrate. (b) Schematic of wound healing assay with defined model parameters relative to wound edge. Experimental results of (c) negative velocity in the *x* direction, (d) traction in the *x* direction, and (e) integral of traction, $\int T_{x}dx$. The slopes of $\int T_{x}dx$ over distance were not statistically different as indicated by the 95% confidence interval on the ratio of slopes which was [0.84, 1.8], spanning unity. Lines in (c)–(e) represent means over multiple fields of view (vehicle control n=5, HB-EGF n=10); shading represents standard deviation. Model results when varying dimensionless traction magnitude $\zeta /(\xi v_{0})$ for (f) active traction, (g) velocity, (h) total traction, and (i) stress. Values of $\zeta /(\xi v_{0})$ used were 1.1, 1.65, 2.2, 2.75, and 3.3. Model results when varying traction decay length ℓ/L for (j) active traction, (k) velocity, (l) total traction, and (m) stress. Model results for two conditions: ℓ/L=0.33, $\zeta /(\xi v_{0})=1.1$ and ℓ/L=0.5, $\zeta /(\xi v_{0})=2.2$, for (n) active traction, (o) velocity, (p) total traction, and (q) stress. All model results are dimensionless as described in the text. Dotted lines in stress plots (e), (i), (m), and (q) represent locations of the maxima of the curves; slopes were computed for all data to the right of each maximum. In panels (i), (m), and (q), numbers by curves refer to the normalized slope of the curve. Slopes were normalized by the magnitude of slope in the baseline case.

The model we use treats the cell monolayer as an active viscous continuum that interacts with the substrate.^30,31^ The model has effectively captured the relationship between force and motion for both expanding cell monolayers and linear chains of cells. For our study, we use the equations of the more recent study,^31^ which presents the minimal essential equations, and, as our results will show, the minimalist approach is sufficient to match our experimental data. A sketch of the model geometry is shown in Fig. 4(a), which shows a side view of the cell monolayer having width 2*L* on a substrate. The model is based on physics and uses force equilibrium as its core principle. In the model, cell velocity *v* is coupled to stress σ through a constant viscosity η according to σ=ηdv/dx. Force equilibrium then gives a balance between gradients in stress and the cell-to-substrate traction *T* according to

$$T=\eta \frac{d^{2}v}{dx^{2}}.$$ (1)

Inertial terms are negligible in this system,^7^ and hence do not appear in the equilibrium equation above. The traction *T* in Eq. (1) is the same as the traction measured in the experiments; namely, it is the traction applied by the cells to the substrate, and it varies over space. A complication is that within a cell monolayer, there are two contributions to the traction. First, active force generation can produce a component of the traction that propels the cells into free space. We call this the active traction and use the symbol $T_{a}$. Second, a viscous drag between the cells and substrate can impede the motion and produce a component in the direction opposite to $T_{a}$. Consistent with many other models,^7^ we assume that the viscous drag is given by ξv, where ξ is a cell–substrate friction coefficient. The traction *T* is then a combination of the active traction $T_{a}$ and the drag term ξv, resulting in the equation:

$$T=\xi v-T_{a}.$$ (2)

Hereafter, we use the word “traction” to mean *T*, which is the sum of the viscous drag and the active term, and we use the words “active traction” to mean $T_{a}$, which contributes to the traction *T* but, depending on the magnitude of ξv, $T_{a}$ may differ substantially from *T*. Finally, the functional form for $T_{a}$ must be chosen. As in a prior study,^31^ we chose the function

$$T_{a}=\zeta \frac{\text{ sinh }(x/\ell )}{\text{ sinh }(L/\ell )},$$ (3)

where ζ represents the magnitude of active traction at the edge of the cell layer, *x* is the position, *L* is half of the cell layer length, and ℓ is a fitting constant that quantifies how quickly the active traction decays over distance into the bulk of the cell monolayer. The hyperbolic sine is used to match experimental observations, namely, that the magnitude of active traction is maximal at the edges of the cell layer (i.e., at x=±L).^31^

We solved Eqs. (1)–(3) together to quantify the velocity *v*, active traction $T_{a}$, and traction *T* depending on the model parameters, which are the monolayer viscosity, η, the cell–substrate friction coefficient, ξ, the magnitude of active traction at the edge of the monolayer, ζ, and the decay length of active traction, ℓ. In the following, we will work in dimensionless units, as follows. Units for length are scaled by *L*. Units for traction are scaled by $\xi v_{0}$, where $v_{0}$ is the scaling for velocity, and it is defined as the magnitude of cell speed at the edge of the monolayer in the baseline model case [ $\zeta /(\xi v_{0})=1.1$, ℓ/L=1/3]. Finally, the viscosity is scaled by $\xi L^{2}$. The dimensionless equations are presented in Appendix B. Following the nondimensionalization, the model has only three parameters: the dimensionless viscosity $\eta /(\xi L^{2})$, the dimensionless active traction $\zeta /(\xi v_{0})$, and the dimensionless decay length of active traction ℓ/L. As this study is interested primarily in the relationship between traction and motion, the viscosity $\eta /(\xi L^{2})$ was set to a single value that provided adequate fitting for all simulations.

To set expectations for the model results, the experimental data were reexamined. First, we note that the experimental variable for position, *d*, is in the opposite direction as the model variable, *x*, which is shown in Fig. 4(b). We plotted negative velocity $-v_{x}$, traction $T_{x}$, and stress ( $\int T_{x}dx$) against position *d* for d>0 [Figs. 4(c)–4(e)]. We make the following four observations, which will form the basis for comparison between the experiments and the model. First, velocity $-v_{x}$ was a factor of ≈2 greater for HB-EGF compared to vehicle control [Fig. 4(c)]. Second, the traction $T_{x}$ at the edge of the cell layer (at d=0) was greater for HB-EGF compared to vehicle control [Fig. 4(d)]. Additionally, $T_{x}$ for d≳50 *μ*m was approximately equal for the two curves [Fig. 4(d)]. Due to force equilibrium, the value of traction in Fig. 4(d) is equal to the slope of the curves of stress in Fig. 4(e), meaning that the slopes of the curves for vehicle control and HB-EGF are approximately equal for d≳50 *μ*m. To verify this observation quantitatively, we quantified the slopes of stress over position in the range starting at the location where stress was a maximum and ending at d=300 *μ*m. 95% confidence intervals of the ratio between slopes for HB-EGF and vehicle control show no significant difference between treatments ([0.84, 1.8], bootstrap). In contrast to the slopes of stress over distance, the magnitudes of stress differed between cells treated with vehicle control and HB-EGF [Fig. 4(e)]. Hence, the third and fourth points for comparison between experiments and model are the magnitude of stress (which was larger for HB-EGF compared to vehicle control) and the slope of stress over distance (which was not different between HB-EGF and vehicle control).

To begin our simulations with the model, we defined a baseline case that matched the vehicle control experiments with parameters: $\zeta /(\xi v_{0})=1.1$, ℓ/L=1/3, and $\eta /(\xi L^{2})=1.1\times 10^{-3}$. We additionally chose to plot the model results in the range −1≤x/L≤−2/3, which approximately matches the experiments; in Fig. 11, Appendix C, we show that the exact range chosen does not substantially affect the model results. Given the observation that treatment with HB-EGF increased traction at the leading edge [Fig. 4(d)], we began our study of the model by varying the magnitude of active traction at the leading edge, $\zeta /(\xi v_{0})$, while keeping constant the decay length, ℓ/L. For this, we defined the active traction decay length to be ℓ/L=1/3 and used five values of $\zeta /(\xi v_{0})$: 1.1, 1.65, 2.2, 2.75, and 3.3. In response to increased $\zeta /(\xi v_{0})$, the active traction [Fig. 4(f)], velocity [Fig. 4(g)], traction [Fig. 4(h)], and stress [Fig. 4(i)] all increased monotonically. Comparing to experimental results, increasing $\zeta /(\xi v_{0})$ matched the effect of HB-EGF on the traction at the edge of the cell layer [Figs. 4(d) and 4(h)]. However, increasing $\zeta /(\xi v_{0})$ caused a more strongly negative traction within the bulk of the cell layer [i.e., for x/L≳−0.9 in Fig. 4(h)], which is evident most clearly in slopes of the curves of stress, which became more strongly negative with increasing $\zeta /(\xi v_{0})$ [Fig. 4(i)]. This result disagrees with the experimental observation that the slopes of stress were the same between vehicle control and HB-EGF [Fig. 4(e)], indicating that changing only the magnitude of active traction does not capture the effects of HB-EGF.

Next, we considered how the traction decays over distance from the edge of the cell monolayer. In the experimental data [Fig. 4(d)], it appears that the traction decayed to zero over a longer distance for cells treated with HB-EGF compared to vehicle control. This observation suggests that HB-EGF may facilitate intercellular coordination of the traction, which is reminiscent of the highly coordinated motion observed in the plots of cell trajectories in Fig. 1(f). To study the decay of traction, we ran a parameter study of the traction decay length ℓ/L, using values of 0.33, 0.4, 0.5, 0.67, and 1. Note that larger values of ℓ/L indicate a longer distance over which the active tractions decay, as illustrated in Fig. 4(j), which shows that increasing ℓ/L leads to a slower spatial decay of active traction. Similarly, increasing ℓ/L causes the velocity to decay more slowly. In other words, as ℓ/L is increased, cells further into the bulk migrate faster [Fig. 4(k)]. Because the total traction *T* is equal to the difference between two quantities, namely, ξv and $T_{a}$ [Eq. (2)], the effect of ℓ/L on the total traction is more complex. Near the edge of the monolayer, larger ℓ/L led to a smaller value of traction, whereas within the bulk, larger ℓ/L caused a greater value of traction [Fig. 4(l)]. The effects of ℓ/L can also be examined by studying the curves of stress, wherein greater values of ℓ/L tend to reduce the magnitude of stress that builds up within the cell layer [Fig. 4(m)].

So far, the simulations have shown that increasing active traction magnitude $\zeta /(\xi v_{0})$ increased the total traction and speed of migration while also changing the slope of stress over distance, whereas increasing the active traction decay length ℓ/L changed the slope of stress over distance with a minimal effect on the traction and speed of migration. On their own, neither parameter study matched the four observations from the experiments listed above. We next hypothesized that to match the experimental observations for HB-EGF, both $\zeta /(\xi v_{0})$ and ℓ/L would need to increase. Compared to the baseline case [ $\zeta /(\xi v_{0})=1.1$; ℓ/L=0.33], we found that increasing $\zeta /(v_{0}\xi )$ to 2.2 and ℓ/L to 0.5 provided a good match to the experimental results for HB-EGF-treated cell monolayers. Specifically, the velocity was approximately a factor of two greater [Fig. 4(o)], the traction at the edge of the cell layer was a factor of 1.4× greater [Fig. 4(p)], the magnitude of stress was greater, and the slope of stress over distance was unchanged [Fig. 4(q)] compared to baseline, all of which matched the four observations described above. Additionally, for HB-EGF, $T_{x}$ crossed zero slightly to the right of the vehicle control case [Fig. 4(d)], which is also matched in the simulations [Fig. 4(p)]. Although perfect quantitative agreement is not present, the agreement between experiments and simulations is strong, especially considering the simplicity of the model. In summary, the model captured effects of HB-EGF with a combination of greater magnitude of active traction [greater $\zeta /(\xi v_{0})$] and active tractions that were distributed further into the bulk of the cell layer (greater ℓ/L).

### Effect of HB-EGF on the cytoskeleton

From the finding that matching the effect of HB-EGF required increasing two variables in the model [ $\zeta /(\xi v_{0})$ and ℓ/L], we can make two predictions. The first is that the magnitude of active traction was larger in response to treatment with HB-EGF. This prediction can be verified by inspecting the magnitude of traction at the edge of the cell layer, which was clearly increased in response to HB-EGF [Fig. 4(d)]. The second prediction is that the decay length of active traction increased in response to HB-EGF. This prediction cannot be tested directly from our traction data, because the traction measured in our experiments is the total traction *T*, which, as shown by Eq. (2), has contributions from both the active traction and the cell–substrate friction coefficient. As a different indicator of the active traction, we studied the structure of the cytoskeleton near the edge of the cell monolayer and within the bulk, expecting to observe differences that could indicate HB-EGF causing active traction to propagate further into the bulk. We defined edge positions as d≤50 *μ*m, which was approximately 2 cell lengths and corresponded to the positions wherein $T_{x}$ was positive [Fig. 4(d)]. Bulk regions were defined as being 200–300 *μ*m from the edge, which is substantially away from the edge yet within the field of view used to measure cell velocities and tractions. We fixed cells at t=5 hr after treatment with HB-EGF or vehicle control, which was chosen as it is approximately halfway through the time-lapse experiments. We then fluorescently stained for F-actin to image the basal stress fibers, which are an indicator of force generation, and for the focal adhesion protein vinculin, which is an indicator of force transmission to the substrate.

**FIG. 5.**
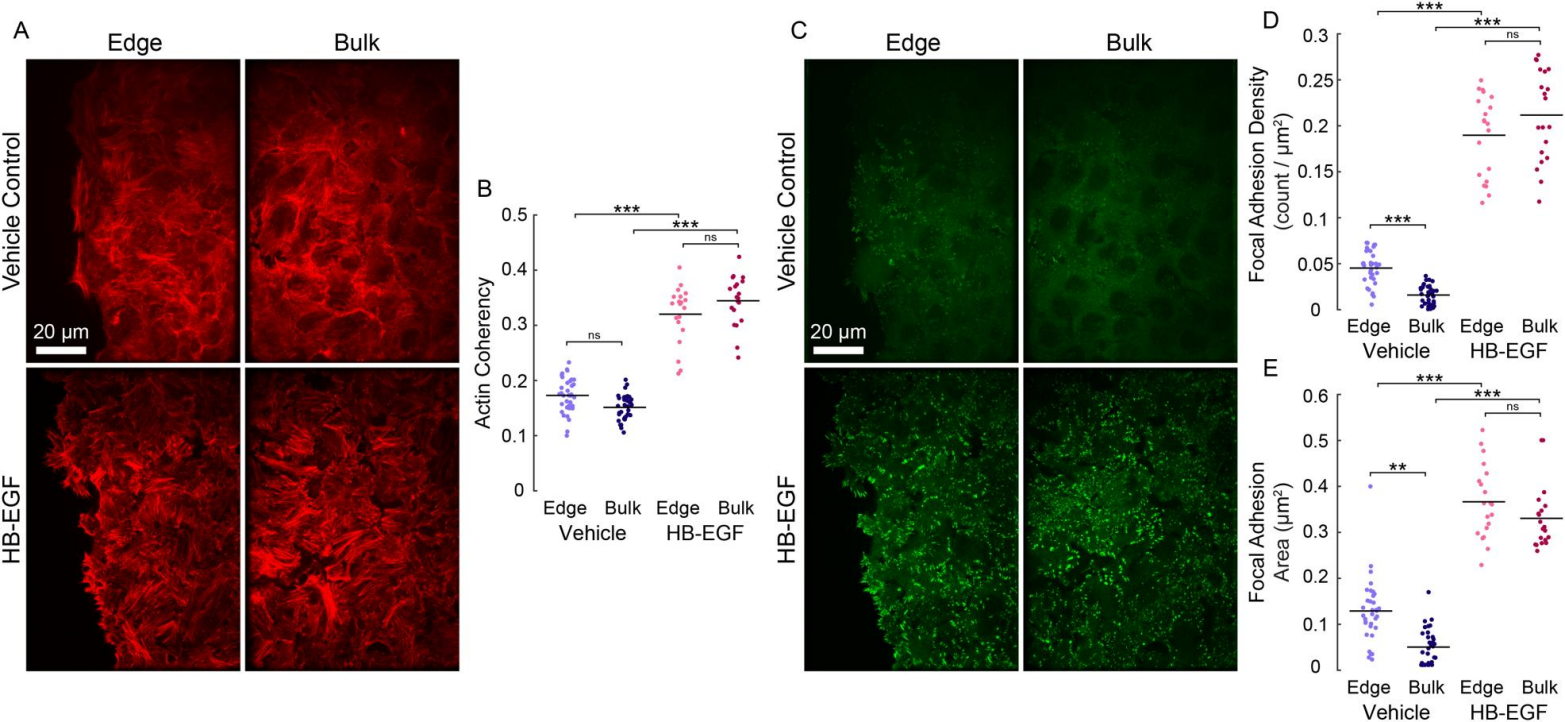
Fluorescent imaging for actin and focal adhesions in expanding cell monolayers. (a) Confocal images of the basal side of HaCaT cells stained for F-actin with vehicle control (top row) or HB-EGF (bottom row). (b) Scatter plot of the coherency of actin stress fiber orientation. (c) Confocal images of HaCaT cells stained for vinculin. (d) Scatter plot of focal adhesion density for vinculin stained monolayers treated with vehicle control and HB-EGF. (e) Scatter plot of average area of each focal adhesion. For all analysis, expanding monolayers were fixed at t=5 hr post-treatment and barrier removal. In all scatter plots, each point is a mean over a field of view, black bars are means over all fields of view. Statistical results of panels in (b), (d), and (e) are located in Appendix D.

First, we studied the images of F-actin, reasoning that because actin intensity is correlated with cell migration and mechanosensing,^32–36^ it would be an indicator of active force production by the cells. Qualitative observations show that the stress fibers displayed greater alignment and clarity in monolayers treated with HB-EGF [Figs. 5(a) and 12, Appendix C]. These observations are consistent with the greater rms traction in response to HB-EGF [Fig. 3(g)]. Notably, the elevated stress fibers in response to HB-EGF existed both at the edge of the cell monolayer and within the bulk, which is reminiscent of the model prediction that the active traction propagated further into the bulk in response to HB-EGF. To quantify the stress fibers, we quantified the local orientation of contrast in the images and calculated the coherency, which is an indicator of local alignment of contrast patterns in an image; it ranges from 0 to 1, with 0 indicating no alignment and 1 indicating strong alignment of contrast patterns along a single orientation. Given the small width of the stress fibers, we calculated coherency in small windows, of size ≈1×1 *μ*m^2^, and repeated this procedure for windows covering the entire cell monolayer. The coherency was then averaged over each field of view, which confirmed the qualitative observations, namely, the coherency was greater for HB-EGF-treated monolayers compared to vehicle control, and there was no reduction in coherency in the bulk compared to the edge [Fig. 5(b)].

In the images of vinculin, there were punctate spots indicating focal adhesions, which were more frequent and larger in size in the images of cells treated with HB-EGF compared to vehicle control [Fig. 5(c)]. Differences were most pronounced for bulk cells in vehicle control conditions, which had very few focal adhesions. To quantify these observations, we first quantified the focal adhesion density by counting the number of local maxima in each image and divided by the area of the image occupied by the cell monolayer. Under vehicle control conditions, the focal adhesion density was lower in the bulk compared to the edge [Fig. 5(d)]. Additionally, in response to HB-EGF, the focal adhesion density was greater than in vehicle control and also was unchanged between bulk and edge [Fig. 5(d)]. We also quantified the average area of each focal adhesion. In vehicle control conditions, focal adhesions at the edge had greater area than in the bulk. In the HB-EGF-treated monolayers, focal adhesion areas were approximately the same between edge and bulk, with both being larger than their vehicle control counterparts [Fig. 5(e)]. Given that focal adhesions transmit cellular forces to the substrate,^37–39^ these observations can be compared to the two model predictions, which are both related to tractions between the cells and substrate. At the edge of the cell monolayer, the elevated focal adhesion density and area in response to HB-EGF are consistent with the increased active traction strength ζ predicted by the model in response to HB-EGF. Within the bulk, the reduced focal adhesion density and area in vehicle control conditions are consistent with the faster decay of active tractions in vehicle control conditions predicted by the model. Finally, the observation that focal adhesion density and size were the same at the edge and within the bulk of HB-EGF-treated monolayers is consistent with the slow decay of active traction in response to HB-EGF predicted by the model.

In summary, the results demonstrate that HB-EGF treatment led to increased alignment and clarity of basal F-actin stress fibers as well as increased focal adhesion density and size, supporting the model prediction. Specifically, the images of basal F-actin showed greater contrast of stress fibers in HB-EGF-treated monolayers, which is consistent with greater cell contractility and monolayer stress. In images of focal adhesions, equal focal adhesion density and size across both the edge and bulk regions in response to HB-EGF suggests that HB-EGF strengthened mechanical coupling throughout the monolayer, in turn causing more cells within the bulk to produce active tractions that propel the collective motion of the monolayer. Therefore, HB-EGF-induced differences in motion can be attributed to maturity of basal F-actin, allowing cells to generate or sustain more active contractility and uniform focal adhesion density and size across HB-EGF-treated monolayers as well as increased focal adhesion localization. This result is consistent with our observation that the stress was greater in response to HB-EGF treatment compared to vehicle control [Fig. 3(o)]. Together, these findings both validate the predictions derived from the model and show how HB-EGF led to a redistribution of the force generation machinery across an expanding monolayer.

## DISCUSSION

In this study, we investigated the relationship between cell forces and collective migration in a model system for wound healing. As a perturbation to the collective migration, we used HB-EGF, which stimulated PLC, resulting in faster and straighter cell migration. Our results also showed that HB-EGF increased the traction applied by the cells to the substrate. To decipher exactly how the forces were altered by HB-EGF, we applied a minimal physics-based model for the migrating cell monolayer, which led to the prediction that HB-EGF increased the magnitude of active cell traction and the distance over which active traction propagated into the bulk of the cell monolayer. Subsequent experiments validated these predictions, namely, vehicle control cell monolayers had reduced focal adhesions in the bulk compared to the edge, whereas HB-EGF monolayers maintained an equal number and size of focal adhesions in the bulk compared to the edge. Together, these findings indicate that in response to HB-EGF, cells within the bulk produced tractions that propelled the monolayer into the free space. In turn, the collective migration was faster and more coordinated in HB-EGF-treated cell monolayers compared to vehicle control.

Our results support that the effects of HB-EGF on tractions and velocity depend on the activation of PLC. PLC activation leads to the conversion of phosphatidylinositol 4,5-bisphosphate (PIP_2_) to IP_3_, in turn leading to elevated intracellular Ca^2+^ and diacylglycerol (DAG).^40^ These secondary messengers provide multiple pathways by which PLC activity could regulate the observed differences in actin and vinculin. For example, elevated intracellular calcium levels activate myosin light chain kinase (MLCK) to increase cell contraction.^41^ Alternatively, DAG is known to act on protein kinases C and D (PKC and PKD). PKC has been shown to interact with vinculin during the early stages of focal adhesion formation^42^ while PKD has been shown to affect E-cadherin and cofilin.^43^

While the physics-based model used here offered a means of identifying how HB-EGF altered the forces to produce faster collective migration, its minimal design means it has some limitations. Because the model reduces the system to a single spatial dimension, it does not account for variability in migration at the wound edge, which can occur, for example, in the formation of protrusions at the edge of the cell monolayer.^20,29,44,45^ This dimensional reduction offered the advantage that it allowed for clearer isolation of key physical principles. With only three input parameters, the model's simplicity provides a transparent interpretation of the dominant physics within the cell layer. Such minimalism comes at the cost of excluding potentially important nonlinear effects driven by mechanosensitive feedback.^5–8^ These omissions may contribute to imperfect agreement between model results and experimental data. Even so, the model succeeded in capturing essential experimental trends and led to specific predictions about the magnitude and decay rate of active tractions, which were confirmed experimentally. It is also notable that this model and a similar one have been used in prior studies of collective migration and monolayer expansion,^30,31^ which highlights the effectiveness of this model for analyzing monolayer expansion experiments.

A key challenge to relating force and motion in collective cell migration is the inability to separate active forces produced by the cells from passive forces resulting from viscoelastic effects.^18–20^ Here, we approached this challenge by supplementing experiments with a theoretical model to provide insight that would be inaccessible by experiments alone. To match the effects of HB-EGF, it was necessary to increase the model parameters for both the magnitude of active traction and the spatial distribution of active traction. Experimental data were consistent with elevated active traction, as indicated by increased traction at the edge of the cell monolayer [Fig. 4(d)] and increased coherence of actin stress fibers [Figs. 5(a) and 5(b)]. Experiments were also consistent with an elevated spatial distribution of active traction, as indicated by the fact that, in response to HB-EGF, the number and density of focal adhesions were high even in the bulk of a cell monolayer [Figs. 5(c)–5(e)]. This finding supports the idea that in response to HB-EGF, cells further into the bulk of the monolayer produced active tractions that propelled the migration. Although this conclusion is not based on direct measurements of active forces, the consistency of the model predictions and cytoskeletal imaging supports our interpretation.

Our conclusions as to how the forces altered the collective motion are specific to the case of an expanding cell monolayer. In confined 1 mm islands (Fig. 9, Appendix C), HB-EGF also increased the cell speed, but not the magnitude of traction. Additionally, in the confined circular islands, the spatial correlation lengths of both velocity and traction were increased by HB-EGF, which is consistent with a prior study showing that spatially correlated tractions have a large effect on the correlation length of velocity.^16^ In contrast, the spatial correlation length of both traction and velocity was unchanged by HB-EGF in the expanding cell monolayers. Another difference is that the migration persistence was increased by HB-EGF in expanding monolayers but not in confined islands. Given that two differences existed between these experiments—shape and confinement—the relative effects of shape and confinement are not yet clear and would be an interesting topic for future research. One approach to study this question would be to quantify the multiple different characteristic length scales within the cell monolayer. One length scale comes from the correlation of intercellular stresses over space.^15,46^ A second, which we have shown here and previously,^16,29^ is that cell–substrate tractions can be spatially correlated as well. A third length scale is caused by the fact that epithelial cells are not perfectly circular; their slightly elongated shapes lead to alignment between the long axes of neighboring cell bodies, which is referred to as nematic order,^47,48^ and occurs over length scales of ≈100 *μ*m in epithelial cells.^49^ A fourth length scale in this system is the hydrodynamic screening length, which is a length scale that results from the ratio of intercellular viscosity and cell–substrate friction.^7,20,29^ Here, as in a recent study,^31^ we identified yet another means for coordination, which is the decay length of active traction (variable ℓ/L in the theory)—larger values of ℓ/L indicate that cells further into the bulk produce tractions that propel the monolayer outward, implying greater intercellular coordination. Simultaneous quantification of these many length scales, including in monolayers with varying shape and confinement, could offer a new avenue for investigating how cells coordinate motion over space.

In the field of collective cell migration, even though the cell forces can be measured, it is challenging to decouple active and passive contributions to the forces, meaning that from a map of the cellular forces it is not yet possible to predict motion. In this study, we used detailed experiments that measured both forces and motion in combination with a theory that accurately matched the experiments and led to predictions as to how force production affects wound closure rate. We then tested the predictions with subsequent experiments that demonstrated how HB-EGF altered the distribution of active cell forces, in turn leading to faster collective migration. In future work, it will be interesting to explore how general the physics-based theory is by applying it to other cell types and other perturbations of cell forces. It will also be valuable to identify precisely how HB-EGF led to active traction that propagated further into the bulk of the cell monolayer. It may be that the altered active traction was the result of changes in adherens junctions that coordinate intercellular communication. Given that effects of HB-EGF were PLC-dependent, it will be interesting to test whether PLC activation leads to changes in cell–cell junctions to enhance active traction, thereby leading to faster and straighter collective migration.

## METHODS

### Cell culture

Human keratinocyte (HaCaT) cells were used for all experiments. The cells were maintained with Dulbecco's Modified Eagle Medium (DMEM, Corning 10-013-CV) with 10% fetal bovine serum (FBS, Corning 35-010-CV) and 1% penicillin/streptomycin (Corning 30-002-CI) at 37 °C with 5% CO_2_ in an incubator. 1 hr before each experiment, cells were transferred to an experimental medium of DMEM having a low glucose concentration of 1 g/l (Corning 10-014-CV) that was supplemented with 1% FBS (Corning 35-010-CV) and 1% penicillin/streptomycin (Corning 30-002-CI). All passages used for experiments were between 38 and 45.

### Fabrication of polyacrylamide substrates

Polyacrylamide substrates with Young's modulus of 6 kPa were fabricated using previously described methods.^50^ Adaptations from this protocol include the addition of 0.5 *μ*m fluorescent microspheres (Thermo Fisher Scientific, F8812). The sum of water and microsphere solution in the polyacrylamide gels was equal to the volume percentage of water reported previously.^50^ Substrates were fabricated on glass-bottom dishes (Cellvis, Mountain View, CA, USA, D35-20-1.5-N). Before the addition of the substrate, the dishes were hydrophobically treated using 0.3% weight/volume (w/v) 3-(Trimethoxysilyl)propyl methacrylate 98% (Acros Organics 216551000) and 0.2% (w/v) acetic acid. 30 min after the addition of the hydrophobic treatment, the dishes were soaked in de-ionized (DI) water for two rounds of 15 min and then left face down to dry overnight. The use of pressurized N_2_ was occasionally used to expedite the drying process. 20 *μ*l of unpolymerized polyacrylamide solution was pipetted onto each treated glass dish. A coverslip (18 mm diameter circle) was then placed on top of each polyacrylamide solution. The gels were then centrifuged upside down for 14 min at 750 rpm. This produces a single layer of fluorescent microspheres on the top surface of the substrate. After centrifugation, DI water was added, and the dishes were stored overnight at 4 °C, allowing the substrate to swell. After the substrate swelled, the coverslips were removed.

### Functionalization of polyacrylamide substrates

For wound healing experiments, the substrates were functionalized by first treating with 50 mg/ml of the covalent cross-linker sulfo-SANPAH (Pierce Biotechnology, Waltham, MA), which was irradiated with ultraviolet light for 16 min and then rinsed with HEPES buffer. Next, 500 *μ*l of 0.01 mg/ml type I rat tail collagen (Corning) was pipetted over the substrate and left at 4 °C overnight prior to cell seeding.

In one set of experiments, shown in Fig. 9 of Appendix C, 1-mm diameter confined cell islands were used. To micropattern the confined islands, polydimethylsiloxane (PDMS, Sylgard 184, Dow Corning) was poured into a Petri dish to a thickness of ≈1 mm and left to cure overnight at 60 °C. The next day, 16 mm diameter circle masks were cut out of the PDMS sheet using a hollow punch. A 1 mm biopsy punch was used to create 6–9 evenly spaced 1 mm holes in each PDMS circle, creating masks for micropatterning. The masks were left overnight at room temperature in 4% Pluronic F-127 (Sigma-Aldrich) to prevent adhesion of collagen I to the masks. The masks were washed three times in phosphate-buffered saline (PBS) and left to dry. Water was aspirated from the dishes containing polyacrylamide substrates. Tweezers were used to apply one mask on each substrate. 3 ml of PBS was added to each dish in which a 200 *μ*l pipet was used to remove air bubbles from the 1 mm holes. Finally, sulfo-SANPAH and collagen I were used as described above to functionalize the substrate through the 1 mm holes. A detailed step-by-step procedure describing the micropatterning process is available online.^51,52^

### Magnetic barriers for monolayer expansion experiments

In order to confine cells to create a wound healing geometry without damaging the underlying substrate, magnetic physical barriers were used.^29,53^ To create these barriers, PDMS (Sylgard 184) was mixed with 200 mesh iron powder (Alfa Aesar) at a concentration of 200 mg/ml. This solution was then added to a plastic Petri dish and left to cure overnight at 60 °C. The resulting sheet of PDMS iron filament mixture was cut into 15 mm by 4 mm rectangles. In a new Petri dish, a layer of PDMS without iron filaments was poured, and the cut rectangles were placed into this new dish. PDMS was then poured over the strips, thus coating the iron filament strips with pure PDMS to prevent oxidation when added to cell culture medium. The dish was once again cured overnight at 60 °C then cut into rectangular strips. The strips were treated with 4% Pluronic F-127 overnight at room temperature to prevent adhesion of collagen or cells to the barrier.

**FIG. 6.**
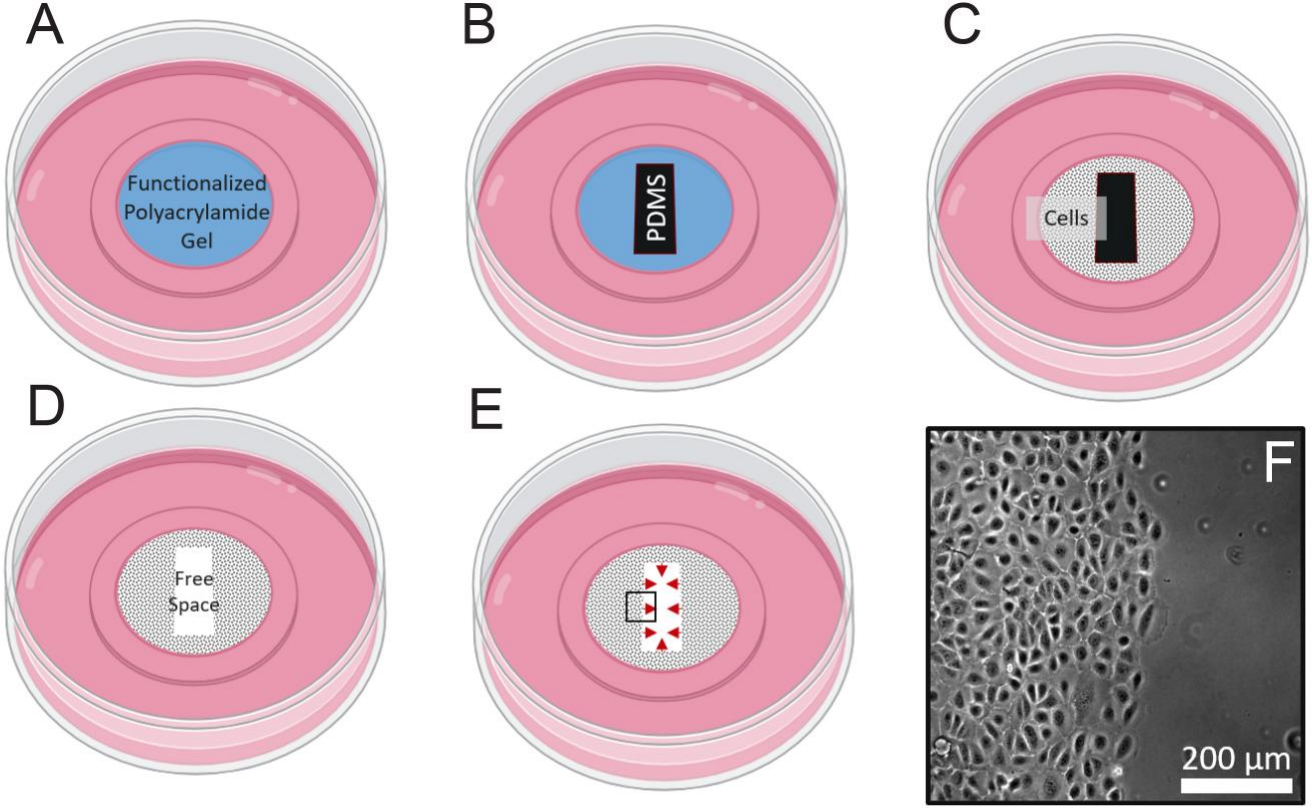
Schematic of cell monolayer expansion assay methodology. (a) Glass-bottom dish with functionalized polyacrylamide gel. (b) PDMS strip with iron filaments held in place by a magnet under the dish. (c) Cells are seeded around the PDMS strip. (d) PDMS strip is removed, creating free space. (e) Cells migrating into free space; the black box represents the field of view in panel (f). (f) Representative phase contrast image of cells in one field of view.

**FIG. 7.**
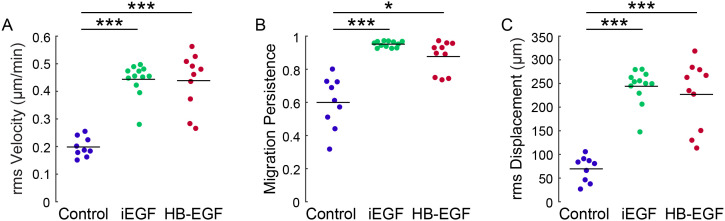
Collective migration in response to HB-EGF was similar to cells on substrates with immobilized EGF (iEGF) in expanding cell monolayers. EGF was immobilized to the surface of polystyrene tissue culture plates using sulfo-SANPAH as previously described.^24^ For control surfaces and HB-EGF conditions, wells were subjected to the same process using 20 mM HEPES. After surface treatment, HaCaT keratinocytes were plated and cultured to confluency inside an Ibidi culture inset (Catalog No. 80209; Ibidi GmbH, Madison, WI, USA) as described.^24^ The inset was lifted and media changed to 500 *μ*l serum-free media containing vehicle (0.1% BSA, used with control or iEGF surfaces) or 60 ng/ml HB-EGF. Cells were imaged every 15 min and analyzed as in the main text. Scatter plots of (a) rms velocity, (b) migration persistence, and (c) rms cell displacement from t=1 to 11 hr after barrier lifting. In all scatter plots, each point is an average over an independent field of view; the black bars represent the mean over all fields of view. Conditions were compared using Dunn–Šidák *post hoc* analysis; results from the statistical test can be found in Appendix D.

**FIG. 8.**
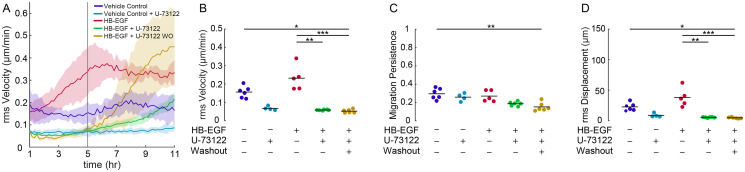
Results of U-73122 treatment on expanding cell monolayers treated with vehicle control and HB-EGF. (a) Rms velocity over t=1–11 hr. Lines are means over multiple fields of view; shaded areas represent standard deviations over multiple fields of view. The vertical line at t=5 hr indicates the point at which the medium was changed from a medium containing HB-EGF + U-73122 to a medium with only HB-EGF in the HB-EGF + U-73122 washout (WO) group. Scatter plots of (b) rms velocity, (c) migration persistence, and (d) rms displacement. Each dot represents an average over a field of view before washout ( t=1–5 hr); black bars represent averages over all fields of view. Statistical results are located in Appendix D.

**FIG. 9.**
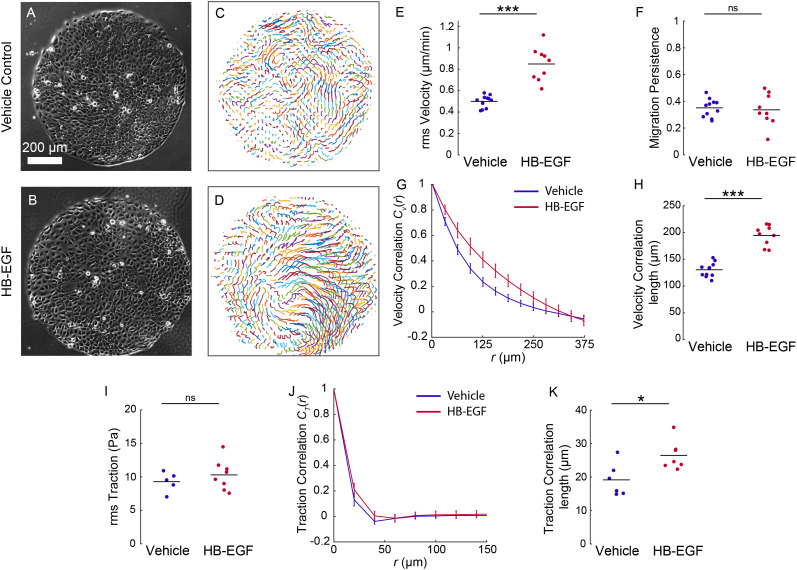
Using a separate approach, cell velocity and traction were measured in confined islands. Phase contrast images of HaCaT cells confined to islands treated with (a) vehicle control and (b) HB-EGF. (c) and (d) Representative cell trajectories computed using FIDIC for cell islands treated with vehicle control and HB-EGF; trajectories shown correspond to t=1–4 hr post-treatment. (e) Scatter plot of rms velocity ( $p=1.9\times 10^{-4}$). (f) Migration persistence for vehicle control and HB-EGF treatment ( p=0.94). (g) Spatial correlation of the velocity vector. Error bars represent standard deviation over multiple fields of view for each treatment. (h) Velocity correlation length ( $p=1.9\times 10^{-4}$). (i) Scatter plot of rms traction ( p=0.93). (j) Spatial correlation of traction. (k) Traction correlation length ( p=0.035). For all scatter plots, each point is an average over an independent field of view, and the black bar represents the mean value over all fields of view.

**FIG. 10.**
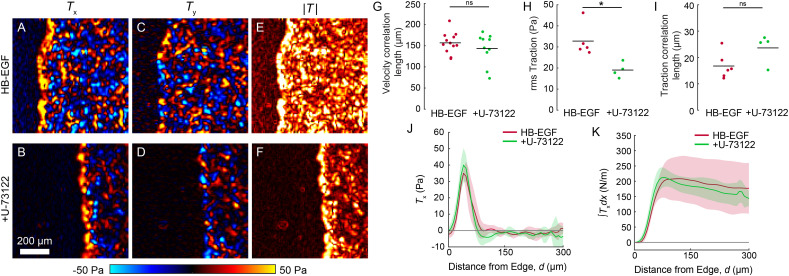
Force production and velocity correlation in expanding cell monolayers treated with HB-EGF and HB-EGF + U-73122, which inhibits PLC. Color maps of traction in the (a) and (b) *x* and (c) and (d) *y* directions as well as (e) and (f) the magnitude of traction (|*T*|). Note that although tractions at the leading edge are both bright in plots of $T_{x}$, bulk $T_{x}$ and $T_{y}$ are dimmer upon treatment with U-73122. This is most clear in panels (e) and (f) where the region behind the bright leading edge is dimmer in the U-73122-treated monolayer. (g) Velocity correlation length, showing no difference between HB-EGF and HB-EGF+U-73122 ( p=0.62). (h) Plots of rms traction quantify the visual differences seen in the color maps, where upon inhibition the rms traction decreases ( $p=1.6\times 10^{-2}$). (i) Scatter plots of traction correlation length show no difference upon treatment with U-73122 ( $p=6.7\times 10^{-2}$). (j) Plotting *x* direction force data against distance from the leading edge *d*, there is little to no effect of U-73122 in the curve of $T_{x}$ when averaged over t=1–11 hr. (k) Results of $\int T_{x}dx$ as a function of distance from the edge *d* also showed little change when averages over t=1–11 hr between HB-EGF and HB-EGF + U-73122.

**FIG. 11.**
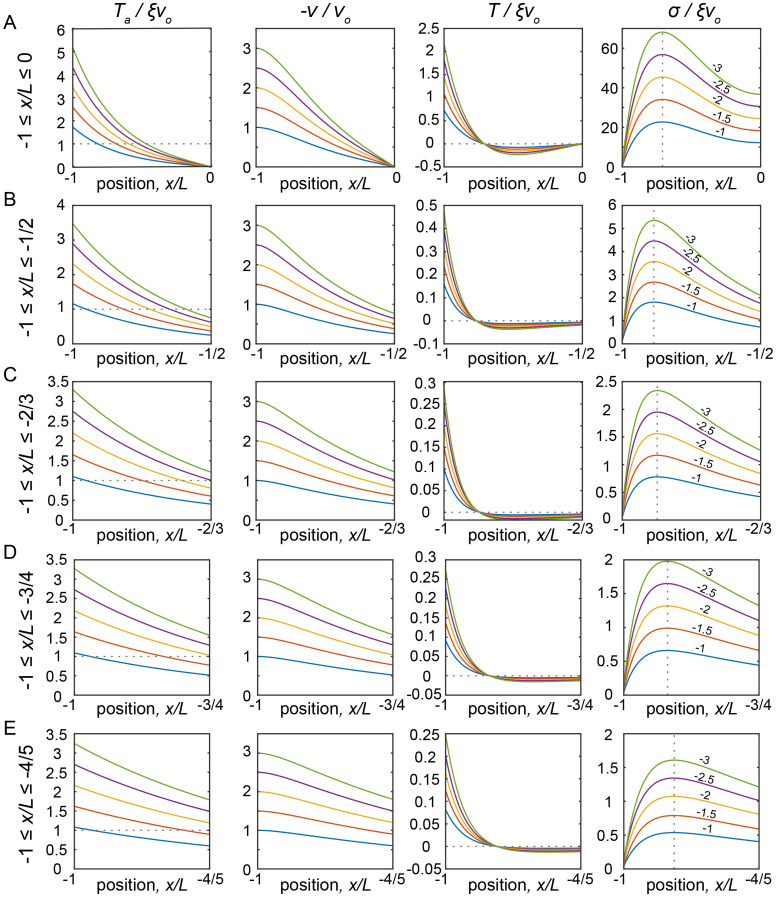
To show that the choice in range of position x/L used for plotting the model results does not substantially change the interpretation, we ran model simulations with varying the chosen position range and model variables. In each plot, the lines indicate different values of ζ, which mimic those in Figs. 4(f)–4(i). Each row represents model results under the following conditions: (a) −1≤x/L≤0, $\zeta /(\xi v_{0})=[1.38,2.05,2.74,3.42,4.10]$; (b) −1≤x/L≤−1/2, $\zeta /(\xi v_{0})=[1.10,1.67,2.22,2.78,3.33]$; (c) −1≤x/L≤−2/3, $\zeta /(\xi v_{0})=[1.10,1.65,2.20,2.75,3.30]$; (d) −1≤x/L≤−3/4, $\zeta /(\xi v_{0})=[1.12,1.68,2.24,2.81,3.37]$; and (e) −1≤x/L≤−4/5, $\zeta /(\xi v_{0})=[1.10,1.66,2.21,2.77,3.32]$. Active traction $T_{a}$ and traction *T* were normalized by passive traction making them unitless. Velocity was normalized by the velocity magnitude of the base case at the edge. Numbers by curves in stress plots refer to the normalized average slope of stress to the right of the dotted line; slopes were normalized by the magnitude of slope in the base case. Dotted lines in the plots for stress, $\sigma /(\xi v_{0})$, represent the maxima of the curve in which the slope was begun to be computed. The results show that choosing different position ranges does not change the resulting trends.

**FIG. 12.**
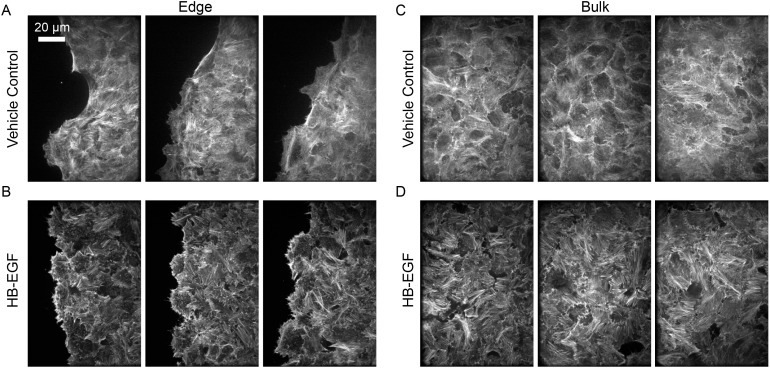
Basal F-actin images of HaCaTs treated with (a) and (c) vehicle control or (b) and (d) HB-EGF in expanding cell monolayers. The cells were fixed at 5 hr after treatment. (a) and (b) show images taken at the edge of the expanding monolayer, while (c) and (d) show images taken in the bulk of the monolayer (200–300 *μ*m from edge). Note increased contrast in actin stress fibers in both edge and bulk treated with HB-EGF vs their vehicle control counterparts, indicating increased cytoskeletal maturation associated with greater contractility and tensile stress.

The barrier strips were then placed upon the functionalized polyacrylamide substrates and held in place by a magnet underneath the dish (Fig. 6, Appendix C). Cells were then seeded around the barrier by pipetting 200 *μ*l of $1.5\times 10^{6}$ cells/ml concentration of cells onto the substrate area not under the barrier. Cells were then incubated at 37 °C and 5% CO_2_ until the cells reached confluence. Once the cells reached confluence, the barrier was removed by first aspirating the cell culture medium and then taking the dish off of the magnet and placing a second magnet over the dish to lift the barrier strip off the gel. Finally, new medium was added to the dishes. Upon removal of the barriers, the cells began to migrate into the free space.

### Biochemical treatments

**FIG. 13.**
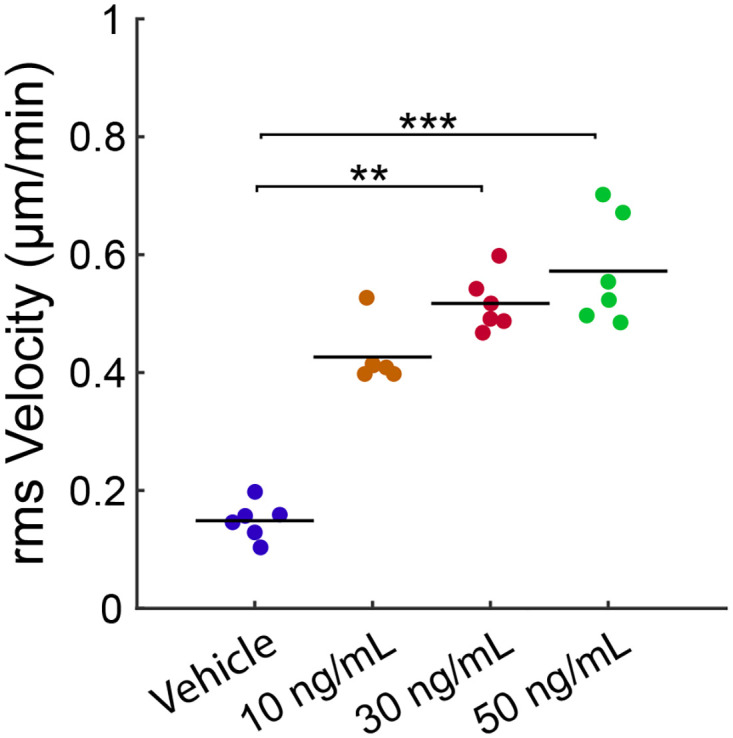
Scatter plot of rms velocity for varying concentrations of HB-EGF in HaCaT monolayers. Each dot represents an average over an independent field of view, black bars represent a mean over all frames of view for each treatment. *p* values from Kruskal–Wallis with Dunn–Šidák correction are as follows: vehicle control–10 ng/ml p=0.39; vehicle control–30 ng/ml $p=7.5\times 10^{-3}$; vehicle control–50 ng/ml $p=7.5\times 10^{-4}$; 10–30 ng/ml p=0.60; 10–50 ng/ml p=0.20; and 30–50 ng/ml p=0.99. Given the *p* value between the 30 and 50 ng/ml HB-EGF concentrations indicates that the rms velocity is not statistically different for these concentrations, we identified 30 ng/ml as a saturation dose. 30 ng/ml HB-EGF was thereby used for all subsequent experiments.

Immediately after removing the barriers, cells were treated with either a vehicle control or HB-EGF. The vehicle was 0.1% bovine serum albumin (BSA, Jackson ImmunoResearch 001-000-162) in PBS. For vehicle control conditions, 100 *μ*l was added to 2 ml of the experimental medium described above. For HB-EGF treatment, recombinant human HB-EGF (R&D Systems 259-HE) was dissolved in 0.1% BSA-PBS prior to dilution in experimental medium. Unless otherwise stated, the HB-EGF concentration was 30 ng/ml; this concentration was chosen based on the results of a dose response experiment, wherein concentrations greater than 30 ng/ml did not significantly alter cell migration speed (Fig. 13, Appendix C). For PLC inhibition, U-73122 (Tocris Bioscience 1268) was added to the treatment media at a final concentration of 5 *μ*M.

### Time-lapse microscopy

**FIG. 14.**
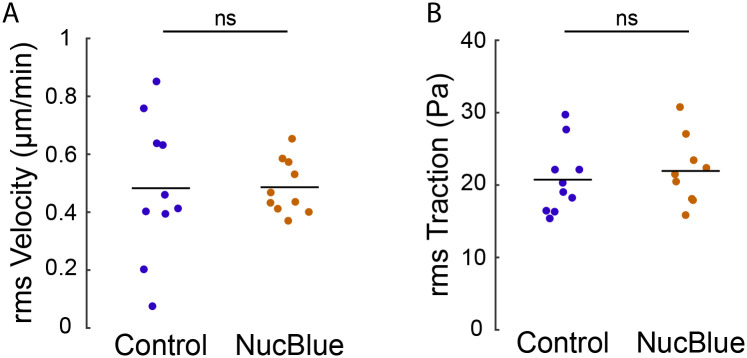
NucBlue live cell stain does not affect rms velocity or rms traction. Scatter plots of HaCaT monolayers in the absence and presence of NucBlue live cell stain for (a) rms velocity ( p=0.97) and (b) rms traction ( p=0.55). Results indicate that there is no difference in rms velocity and rms traction between monolayers treated and not treated with NucBlue, signifying that treatment with NucBlue does not affect motion or force production.

For all time-lapse experiments, cell nuclei were stained using NucBlue Live ReadyProbes Reagent Hoechst 33342 (Thermo R37605). 15 *μ*l of the reagent was added to dishes containing 2 ml cell media, where it was then incubated at 37 °C with 5% CO_2_ for 15 min. The reagent/media mixture inside the dish was then aspirated out and rinsed with warm experimental medium. We verified that the Hoechst treatment did not affect the rms cell velocity or rms cell traction (Fig. 14, Appendix C). Subsequently, the barriers were removed and treatments were added. The time point at which the barriers were removed and the treatments were added was defined as t=0 hr.

The dishes containing migrating cells were placed into a custom-built incubator stage on an optical microscope, where they were kept at 37 °C with 5% CO_2_. After 1 hr (which was necessary for the temperature to equilibrate) time-lapse imaging was performed. A 10× objective with a 0.30 numerical aperture was used on an inverted Eclipse Ti microscope run by Nikon NIS-Elements Ar software (Nikon Instrument). In most imaging, an intermediate magnification changer was used to enhance magnification to 15×. Time-lapse images were taken at intervals ranging from 8 to 12 min over time periods 1–11 hr after barrier removal and HB-EGF treatment. The cells were imaged by phase contrast. A DAPI (360/460 nm) fluorescent channel was used to image the cell nuclei over time, as well as a red (660/680 nm) fluorescent microscope channel for embedded bead imaging.

### Quantification of cell monolayer boundaries, cell velocity, cell trajectories, and migration persistence

**FIG. 15.**
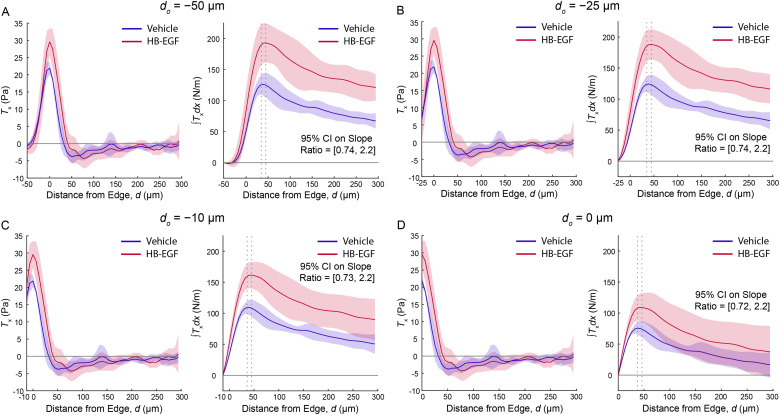
Segmentation of expanding cell monolayers was accomplished using fluorescent images of cell nuclei. As nuclei are located in the center of each cell, the cell edges, including lamellipodia, were located outside the segmentation. To account for this, in the main text, traction and stress were computed and plotted beginning at $d_{0}=-50$ *μ*m. To test the sensitivity of the traction and stress to the choice of the starting value, $d_{0}$, this figure shows results for varying starting values, namely, (a) $d_{0}=-50\;\mu m$, (b) $d_{0}=-25\;\mu m$, (c) $d_{0}=-10\;\mu m$, and (d) $d_{0}=0\;\mu m$. As shown in the figures, as $d_{0}$ is increased, less of the positive traction data is retained, leading to a reduced magnitude of stress ( $\int T_{x}dx$). Despite the difference in magnitude of stress, the main trends remain the same. In particular, $T_{x}$ near the edge of the cell monolayer was greater for HB-EGF compared to vehicle control, and the slopes of stress between vehicle control and HB-EGF were not statistically different, as indicated by the 95% confidence intervals on the ratios of the slopes, which spanned unity. In all plots, lines represent averages over multiple fields of view; shaded regions indicate standard deviation over fields of view.

In both cell islands and wound healing geometries, cell segmentation was performed to identify the area and location of the monolayer. Segmentation was accomplished by taking time-lapse images of nuclei and applying a bandpass filter.^54^ Then, all pixels with intensity above a manually chosen threshold were identified, dilated, and then eroded, yielding a segmentation of the monolayer. Due to the segmentation being based off of cell nuclei, at the monolayer edge lamellipodia may produce tractions outside of the segmented region. Therefore, for wound healing experiments, segmentations were shifted 50 *μ*m into the free space to include all tractions produced by the cells. The choice to use 50 *μ*m did not substantially affect the results, as using different shift distances ( $d_{0}=-50$, −25, −10, and 0 *μ*m) had minimal effects on the trends (Fig. 15, Appendix C).

To compute cell velocities from the images, we used fast iterative digital image correlation (FIDIC)^55^ on the phase contrast images. For all data analyzed, a subset size of 64×64 pix and a spacing of 16 pix were used. Displacements were divided by time between images, resulting in cell monolayer velocities. For cell island experiments, images were collected every 10 min; for monolayer expansion experiments, 8 min.

Cell trajectories were estimated from the cell displacement data as described previously.^56^ Migration persistence was calculated by taking the end-to-end displacement of each cell trajectory and dividing by the total distance the cell traveled over the same time period. This was done for all trajectories, and an average was taken for each field of view.

### Traction force microscopy

During the process of polyacrylamide substrate fabrication described, 0.5 *μ*m fluorescent microspheres were embedded in the top surface of the substrate. Upon completion of the time-lapse microscopy, cells were removed from the substrate using trypsin and 3% (w/v) Triton X-100. Upon removal of the cells, the substrate returned to an unstressed reference state. FIDIC was then used to compute the substrate displacements at each time point with respect to the reference state. Cell–substrate tractions were computed from the displacements using Fourier transform traction cytometry^57^ with the finite substrate thickness accounted for.^12,58^ Images of the cell nuclei were used to find the monolayer boundary by segmentation as described above; only tractions within the monolayer boundary were used for further analysis. Tractions outside of monolayer boundaries were used to quantify the noise of the measurement. Fields of view containing poor-quality traction data were excluded if they demonstrated drift of fluorescent particles causing a noise floor >5 Pa and/or damage to the substrate upon removing cells with trypsin. Due to these exclusion criteria, the number of samples sometimes differed between kinematic and traction data.

### Computation of single-cell traction

**FIG. 16.**
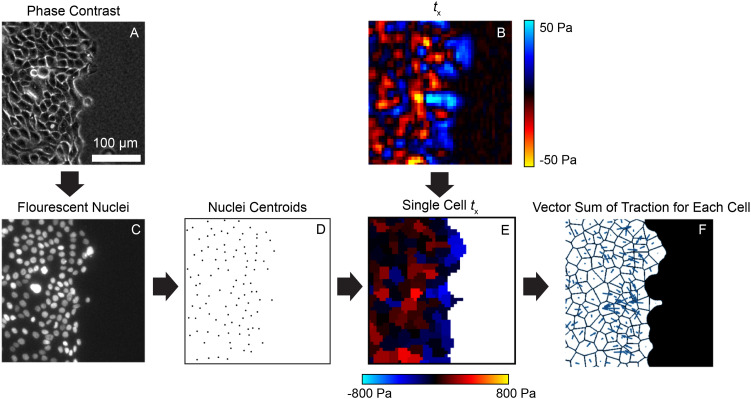
Method for mapping traction to each cell. (a) Phase contrast image of cells in a representative experiment. (b) Cell–substrate traction in the *x* direction applied by the cells in panel (a). (c) Fluorescent nuclei of cells stained using Hoechst. (d) Centroids of nuclei identified using segmentation. A Voronoi tessellation was made using the coordinates of centroids; each polygon in the tessellation is an estimate of the corresponding cell's outline. (e) Provided the tessellation, the traction from panel (b) is summed under the area of each polygon. This process is repeated for traction components in the *y* direction. (f) Given the *x* and *y* components of traction, the net traction vector produced by each cell is computed.

In order to compute the spatial correlation of traction, tractions had to be mapped to individual cells. To do so, we followed a previously published approach,^16^ which is illustrated in Fig. 16 of Appendix C. To begin, the images of fluorescent nuclei were bandpass filtered, and the centroid of each nucleus was tracked over time using particle tracking methods.^54^ For each time point, a Voronoi tessellation was constructed based on the nuclei locations. Vertices outside the monolayer boundary were removed. The tractions under each Voronoi cell were summed in the *x* and *y* directions, resulting in a traction vector sum for each cell.

### Spatial correlation and correlation length

Spatial correlations were computed according to

$$C_{u}(r)=\left\langle \hat{u}(r^{\prime })\cdot \hat{u}(r^{\prime }+r)\right\rangle ,$$ (4)

where $\hat{u}(r^{\prime })$ is the unit vector of the variable of interest at point $r^{\prime }$ in space and $\hat{u}(r^{\prime }+r)$ is the unit vector at a distance r away from $r^{\prime }$. The brackets indicate that the dot products were averaged for all points at distance r from the point of interest $r^{\prime }$ and then averaged again over all points in space, i.e., over all $r^{\prime }$. For computing the velocity correlation, the variable $\hat{u}$ was replaced by the unit vector of velocity $\hat{v}$ in Eq. (4); for computing the traction correlation, it was replaced by the unit vector of single-cell traction $\hat{T}$ (where the procedure for mapping to individual cells is described above and in Fig. 16 of Appendix C). To quantify the correlation lengths from the spatial correlations, the distance over which the spatial correlation decreased to a threshold of 0.2 was quantified, as in prior studies.^16,29,46^

### Fluorescent staining and fixed microscopy

For imaging of focal adhesions and basal F-actin, cells were fixed with 4% paraformaldehyde solution for 20 min. Cells were then washed twice with PBS and stored at 4 °C. Cell membranes were permeabilized by removing the PBS and incubating cells in 0.4% Triton X-100 (Thermo 85111) diluted in PBS for 5 min, followed by washing twice with PBS. A solution of 3% bovine serum albumin (BSA, Jackson ImmunoResearch 001-000-162) was added, and cells were left overnight. Vinculin monoclonal antibody conjugated to Alexa Fluor 488 (Thermo 53-9777-82) was diluted 1:200 in 2% BSA, added to the dishes, and left to stain overnight. The next day, 2 drops/ml of actin ReadyProbes (Thermo R37112) were added and incubated for 30 min at room temperature. Cells were washed twice in PBS and stored at 4 °C before imaging.

For fixed cell imaging, a CSU-X1 spinning disk microscope (Yokogawa) mounted on a Nikon Ti stand was used with a 60× numerical aperture 1.49 oil immersion objective (Nikon) and a Zyla camera (Andor). Images were taken in a z-stack with 1 *μ*m steps for imaging vinculin and actin. Cells at both the edge and bulk of the expanding monolayer were imaged. The image at the cell–substrate interface was selected from each stack for further analysis.

Actin coherency was quantified by applying the FIJI^59,60^ plugin OrientationJ to images of basal F-actin. First, the local orientation was quantified using a window size of 10 pixels (1.08 *μ*m). The coherency was calculated as defined previously.^61^ Values of coherency were analyzed only at locations occupied by the cell monolayer, and they were averaged over each field of view. To quantify focal adhesion density, images of vinculin were analyzed using the Find Maxima function in FIJI,^59,60^ in which a prominence value of 100 was used. To account for images of the monolayer edge that do not take up the entire frame, the number of maxima were then divided by the area of the monolayer in *μ*m^2^, which was quantified by segmentation as described above. The result of this analysis was a focal adhesion density. In order to compute the focal adhesion area, images of vinculin were thresholded so that only the bright focal adhesion remained. Using the “regionprops” function in Matlab, the area of each focal adhesion was computed. The areas were then converted to *μ*m^2^ and averaged over all focal adhesions in each field of view, resulting in the average focal adhesion size for that field of view.

### Statistical analysis

In each experiment, data were obtained over space and time in multiple fields of view, each separated by >1.5 mm in the same dish as well as across different dishes. The average over each field of view was considered an independent measurement for statistical testing. Statistical comparisons were made using a Wilcoxon rank sum test or, for multiple comparisons, a Kruskal–Wallis test with Dunn–Šidák correction. Statistical significance for all analyses was indicated by asterisks as follows: ${}^{*}p<0.05$, ${}^{**}p<0.01$, and ${}^{***}p<0.001$.

To assess differences in slope of stress between monolayers treated with vehicle control vs HB-EGF, the slope of the curve of $\int T_{x}dx$ over distance was computed by taking the gradient between each data point to the right of the maxima of $\int T_{x}dx$, then averaging over the region. A custom bootstrap analysis was performed on the ratio between slopes of HB-EGF and vehicle control with 10 000 bootstrap samples to quantify the 95% confidence interval.

All experiments in this study were performed at least twice, with trends reported in this manuscript matching for both experimental repeats. Data presented in this manuscript are representative results from one of the experimental repeats.

## Data Availability

The data that support the findings of this study are available from the corresponding author upon reasonable request.

## APPENDIX A: VELOCITY AND TRACTION CORRELATION IN CONFINED CELL MONOLAYERS

We considered that the high level of persistence in response to HB-EGF in expanding monolayers may have been a result of enhanced intercellular coordination, wherein the cells matched their velocity with that of their neighbors. We previously showed that one underlying cause of coordinated cell motion is that neighboring cells tend to produce tractions on the substrate that point in the same direction.^16,29^ Motivated by this finding, we quantified the spatial correlation lengths of cell velocity and traction in response to HB-EGF. As shown in the main text for expanding monolayers, there was no difference in the spatial alignment of traction between vehicle control and HB-EGF-treated monolayers (Fig. 3), despite the different migration speed and persistence (Fig. 1). To investigate this apparent disagreement further, we decided to test the effects of different boundary conditions, and we performed one set of experiments in 1 mm confined islands [Figs. 9(a) and 9(b)]. In the confined islands, cell trajectory maps indicated faster migration and strong spatial coordination of velocity in response to HB-EGF [Figs. 9(c) and 9(d)]. The rms velocity was a factor of ≈2 larger in cell monolayers treated with HB-EGF compared to vehicle control [Fig. 9(e)]. Interestingly, the migration persistence was approximately equal in vehicle control and HB-EGF-treated monolayers [Fig. 9(f)], which is likely due to the fact that the circular geometry confines the cells, thereby restricting them to migrate in curved paths [Fig. 9(d)]. We also quantified the spatial correlation of cell velocity $C_{v}(r)$. In response to HB-EGF, the spatial correlation was notably larger than vehicle control [Fig. 9(g)]. For vehicle control conditions, the velocity correlation length was ≈135 *μ*m, and with HB-EGF, it was ≈190 *μ*m, indicating that the velocity fields were more strongly correlated in HB-EGF treated, confined monolayers.

Next, we quantified the cell–substrate forces using traction force microscopy. Interestingly, despite the difference in rms velocity caused by HB-EGF, there was no difference in rms traction between vehicle control and HB-EGF-treated cell islands [Fig. 9(i)]. Therefore, we considered that HB-EGF stimulation could have altered the spatial coordination of traction. When quantifying the spatial correlation length of traction, it is important to consider how cells produce traction on the substrate. As cells contract against the substrate, they pinch on it, creating traction in opposite directions at either end of the cell.^57,62,63^ As a result, the spatial correlation of the traction map would decay to zero over a distance approximately equal to the size of a single cell. To account for this issue, we performed an enhanced analysis, wherein the traction was mapped to each cell. Because cells apply force to their neighbors in addition to the substrate, the vector sum of traction underneath each cell does not equal zero; this sum of traction quantifies the net force (both magnitude and direction) between each cell and the substrate. We have previously shown that the net traction produced by each is correlated over space and that small changes in traction correlation length have large effects on the spatial correlation of velocity.^16,29^ Therefore, we mapped the distribution of traction to each cell, computed a vector sum, and quantified the spatial correlation $C_{T}(r)$ (see Methods for details). Cells treated with HB-EGF had a slightly greater traction correlation than vehicle control [Fig. 9(j)]. This observation was confirmed by quantifying the correlation length, which was ≈19 *μ*m in vehicle control conditions and ≈28 *μ*m for HB-EGF-treated cell islands [Fig. 9(k)].

Although the change in traction correlation length in confined monolayers upon treatment with HB-EGF may appear modest (from ≈19 to 25 *μ*m), prior work demonstrated that small changes in the traction correlation length can substantially influence the velocity correlation length,^16^ so it is reasonable to infer that in confined monolayers, the traction correlation length had a notable effect on the migration. In expanding monolayers, the traction correlation lengths were ≈27 *μ*m under both vehicle control and HB-EGF treatment. These correlation lengths ( ≈27 *μ*m) are approximately equal to the upper end of the range observed for confined islands ( ≈25 *μ*m). From this, one possible explanation is that the maximal value for traction correlation length for these cells may be in the range of 25–27 *μ*m, meaning that in the case of expanding monolayers, treatment with HB-EGF caused no further increase. As described in the main text, HB-EGF led to other changes in the tractions in expanding monolayers, namely, an increase in the magnitude of active traction and an increase in the number of cells within the bulk of the monolayer that produced active, propulsive traction.

## APPENDIX B: NONDIMENSIONALIZATION OF EQUATIONS IN THE THEORETICAL MODEL

The model was implemented in dimensionless units, which offers multiple advantages, including making the equations scale-independent and enabling comparison between experiments and the model. Here, we show the model equations in dimensionless form. As described in the main text, traction *T* was divided by $\xi v_{0}$ and position *x* was divided by *L*, meaning that Eq. (1) in dimensionless form is

$$\frac{T}{\xi v_{0}}=\frac{\eta }{\xi L^{2}}\frac{d^{2}(v/v_{0})}{d{\left(x/L\right)}^{2}}.$$

From this equation, it is apparent that the dimensionless viscosity is $\eta /(\xi L^{2})$. Equation (2) from the main text was divided by $\xi v_{0}$ to give

$$\frac{T}{\xi v_{0}}=\frac{v}{v_{0}}-\frac{T_{a}}{\xi v_{0}}.$$

Finally, Eq. (3) from the main text in nondimensional form becomes

$$\frac{T_{a}}{\xi v_{0}}=\frac{\zeta }{\xi v_{0}}\frac{\text{ sinh }\left(\frac{x/L}{\ell /L}\right)}{\text{ sinh }\left(\frac{1}{\ell /L}\right)}.$$

## APPENDIX C: SUPPLEMENTARY FIGURES

This appendix contains Figs. 6–16.

## APPENDIX D: p VALUES OF MULTIPLE COMPARISON TESTS

For all statistical tests on multiple comparisons, Dunn–Šidák *post hoc* analyses were implemented following a Kruskal–Wallis test. The results are listed in Tables I–XII.

**TABLE I.** Statistical results for Fig. 2(h) rms velocity in response to U-73122 ( t=5–11 hr).

Group 1	Group 2	*p* value
Vehicle control	Vehicle control + U-73122	0.24
Vehicle control	HB-EGF	0.47
Vehicle control	HB-EGF + U-73122	0.94
Vehicle control	HB-EGF + U-73122 WO	0.86
Vehicle control + U-73122	HB-EGF	0.0014
Vehicle control + U-73122	HB-EGF + U-73122	0.94
Vehicle control + U-73122	HB-EGF + U-73122 WO	0.0063
HB-EGF	HB-EGF + U-73122	0.029
HB-EGF	HB-EGF + U-73122 WO	1.0
HB-EGF + U-73122	HB-EGF + U-73122 WO	0.11

**TABLE II.** Statistical results for Fig. 2(i) migration persistence in response to U-73122 ( t=5–11 hr).

Group 1	Group 2	*p* value
Vehicle control	Vehicle control + U-73122	0.20
Vehicle control	HB-EGF	1.0
Vehicle control	HB-EGF + U-73122	0.022
Vehicle control	HB-EGF + U-73122 WO	0.93
Vehicle control + U-73122	HB-EGF	0.052
Vehicle control + U-73122	HB-EGF + U-73122	1.0
Vehicle control + U-73122	HB-EGF + U-73122 WO	0.92
HB-EGF	HB-EGF + U-73122	0.0037
HB-EGF	HB-EGF + U-73122 WO	0.53
HB-EGF + U-73122	HB-EGF + U-73122 WO	0.48

**TABLE III.** Statistical results for Fig. 2(j) rms displacement in response to U-73122 ( t=5–11 hr).

Group 1	Group 2	*p* value
Vehicle control	Vehicle control + U-73122	0.050 3
Vehicle control	HB-EGF	0.72
Vehicle control	HB-EGF + U-73122	0.43
Vehicle control	HB-EGF + U-73122 WO	1.0
Vehicle control + U-73122	HB-EGF	0.000 42
Vehicle control + U-73122	HB-EGF + U-73122	0.96
Vehicle control + U-73122	HB-EGF + U-73122	0.22
HB-EGF	HB-EGF + U-73122	0.006 9
HB-EGF	HB-EGF + U-73122 WO	0.28
HB-EGF + U-73122	HB-EGF + U-73122 WO	0.88

**TABLE IV.** Statistical results for Fig. 5(b) actin coherency.

Group 1	Group 2	*p* value
Vehicle control—edge	Vehicle control—bulk	0.21
Vehicle control—edge	HB-EGF—edge	$6.0\times 10^{-6}$
Vehicle control—edge	HB-EGF—bulk	$1.5\times 10^{-7}$
Vehicle control—bulk	HB-EGF—edge	$1.6\times 10^{-10}$
Vehicle control—bulk	HB-EGF—bulk	$1.2\times 10^{-12}$
HB-EGF—edge	HB-EGF—bulk	0.99

**TABLE V.** Statistical results for Fig. 5(d) focal adhesion density.

Group 1	Group 2	*p* value
Vehicle control—edge	Vehicle control—bulk	0.001 0
Vehicle control—edge	HB-EGF—edge	0.000 13
Vehicle control—edge	HB-EGF—bulk	$7.2\times 10^{-6}$
Vehicle control—bulk	HB-EGF—edge	$5.8\times 10^{-13}$
Vehicle control—bulk	HB-EGF—bulk	$4.7\times 10^{-15}$
HB-EGF—edge	HB-EGF—bulk	0.99

**TABLE VI.** Statistical results for Fig. 5(e) focal adhesion area.

Group 1	Group 2	*p* value
Vehicle control—edge	Vehicle control—bulk	0.0036
Vehicle control—edge	HB-EGF—edge	$2.3\times 10^{-5}$
Vehicle control—edge	HB-EGF—bulk	$3.2\times 10^{-5}$
Vehicle control—bulk	HB-EGF—edge	$3.4\times 10^{-13}$
Vehicle control—bulk	HB-EGF—bulk	$6.0\times 10^{-13}$
HB-EGF—edge	HB-EGF—bulk	1.0

**TABLE VII.** Statistical results for Fig. 7(a) rms velocity under immobilized EGF and HB-EGF treatment.

Group 1	Group 2	*p* value
Control	iEGF	0.000 65
Control	HB-EGF	0.000 29
iEGF	HB-EGF	0.97

**TABLE VIII.** Statistical results for Fig. 7(b) migration persistence under immobilized EGF and HB-EGF treatment.

Group 1	Group 2	*p* value
Control	iEGF	$3.0\times 10^{-5}$
Control	HB-EGF	0.012
iEGF	HB-EGF	0.38

**TABLE IX.** Statistical results for Fig. 7(c) rms displacement under immobilized EGF and HB-EGF treatment.

Group 1	Group 2	*p* value
Control	iEGF	0.000 28
Control	HB-EGF	0.000 75
iEGF	HB-EGF	1.0

**TABLE X.** Statistical results for Fig. 8(b) rms velocity in response to U-73122 ( t=1–5 hr).

Group 1	Group 2	*p* value
Vehicle control	Vehicle control + U-73122	0.87
Vehicle control	HB-EGF	0.98
Vehicle control	HB-EGF + U-73122	0.11
Vehicle control	HB-EGF + U-73122 WO	0.020
Vehicle control + U-73122	HB-EGF	0.26
Vehicle control + U-73122	HB-EGF + U-73122	0.99
Vehicle control + U-73122	HB-EGF + U-73122 WO	0.81
HB-EGF	HB-EGF + U-73122	0.007 1
HB-EGF	HB-EGF + U-73122 WO	0.000 82
HB-EGF + U-73122	HB-EGF + U-73122 WO	1.0

**TABLE XI.** Statistical results for Fig. 8(c) migration persistence in response to U-73122 ( t=1–5 hr).

Group 1	Group 2	*p* value
Vehicle control	Vehicle control + U-73122	1.0
Vehicle control	HB-EGF	1.0
Vehicle control	HB-EGF + U-73122	0.056
Vehicle control	HB-EGF + U-73122 WO	0.0055
Vehicle control + U-73122	HB-EGF	1.0
Vehicle control + U-73122	HB-EGF + U-73122	0.51
Vehicle control + U-73122	HB-EGF + U-73122 WO	0.14
HB-EGF	HB-EGF + U-73122	0.33
HB-EGF	HB-EGF + U-73122 WO	0.062
HB-EGF + U-73122	HB-EGF + U-73122 WO	1.0

**TABLE XII.** Statistical results for Fig. 8(d) rms displacement in response to U-73122 ( t=1–5 hr).

Group 1	Group 2	*p* value
Vehicle control	Vehicle control + U-73122	0.96
Vehicle control	HB-EGF	0.99
Vehicle control	HB-EGF + U-73122	0.077
Vehicle control	HB-EGF + U-73122 WO	0.009 3
Vehicle control + U-73122	HB-EGF	0.51
Vehicle control + U-73122	HB-EGF + U-73122	0.90
Vehicle control + U-73122	HB-EGF + U-73122 WO	0.48
HB-EGF	HB-EGF + U-73122	0.007 5
HB-EGF	HB-EGF + U-73122 WO	0.000 65
HB-EGF + U-73122	HB-EGF + U-73122 WO	1.0

## References

[1] A. J. Singer and R. A. Clark, N. Engl. J. Med. 341, 738 (1999). 10.1056/NEJM199909023411006 10471461

[2] S. K. Raja, M. S. Garcia, R. R. Isseroff *et al.*, Front. Biosci. 12, 2849 (2007). 10.2741/2277 17485264

[3] I. Pastar, O. Stojadinovic, N. C. Yin, H. Ramirez, A. G. Nusbaum, A. Sawaya, S. B. Patel, L. Khalid, R. R. Isseroff, and M. Tomic-Canic, Adv. Wound Care 3, 445 (2014). 10.1089/wound.2013.0473 PMC4086220 25032064

[4] P. Rousselle, F. Braye, and G. Dayan, Adv. Drug Delivery Rev. 146, 344 (2019). 10.1016/j.addr.2018.06.019 29981800

[5] X. Trepat and E. Sahai, Nat. Phys. 14, 671 (2018). 10.1038/s41567-018-0194-9

[6] B. Ladoux and R.-M. Mège, Nat. Rev. Mol. Cell Biol. 18, 743 (2017). 10.1038/nrm.2017.98 29115298

[7] R. Alert and X. Trepat, Annu. Rev. Condens. Matter Phys. 11, 77 (2020). 10.1146/annurev-conmatphys-031218-013516

[8] S. Banerjee and M. C. Marchetti, Phys. Rev. Lett. 109, 108101 (2012). 10.1103/PhysRevLett.109.108101 23005331

[9] P. Chakraborty, Phys. Fluids 35, 061702 (2023). 10.1063/5.0152826

[10] L. Chen, K. Painter, C. Surulescu, and A. Zhigun, Philos. Trans. R. Soc., B 375, 20190379 (2020). 10.1098/rstb.2019.0379 PMC7423384 32713297

[11] X. Serra-Picamal, V. Conte, R. Vincent, E. Anon, D. T. Tambe, E. Bazellieres, J. P. Butler, J. J. Fredberg, and X. Trepat, Nat. Phys. 8, 628 (2012). 10.1038/nphys2355

[12] X. Trepat, M. R. Wasserman, T. E. Angelini, E. Millet, D. A. Weitz, J. P. Butler, and J. J. Fredberg, Nat. Phys. 5, 426 (2009). 10.1038/nphys1269

[13] J. H. Kim, X. Serra-Picamal, D. T. Tambe, E. H. Zhou, C. Y. Park, M. Sadati, J.-A. Park, R. Krishnan, B. Gweon, E. Millet *et al.*, Nat. Mater. 12, 856 (2013). 10.1038/nmat3689 23793160 PMC3750079

[14] E. Bazellières, V. Conte, A. Elosegui-Artola, X. Serra-Picamal, M. Bintanel-Morcillo, P. Roca-Cusachs, J. J. Muñoz, M. Sales-Pardo, R. Guimerà, and X. Trepat, Nat. Cell Biol. 17, 409 (2015). 10.1038/ncb3135 25812522 PMC4886824

[15] D. T. Tambe, C. Corey Hardin, T. E. Angelini, K. Rajendran, C. Y. Park, X. Serra-Picamal, E. H. Zhou, M. H. Zaman, J. P. Butler, D. A. Weitz *et al.*, Nat. Mater. 10, 469 (2011). 10.1038/nmat3025 21602808 PMC3135682

[16] A. Saraswathibhatla, S. Henkes, E. E. Galles, R. Sknepnek, and J. Notbohm, Extreme Mech. Lett. 48, 101438 (2021). 10.1016/j.eml.2021.101438

[17] M. Vishwakarma, J. Di Russo, D. Probst, U. S. Schwarz, T. Das, and J. P. Spatz, Nat. Commun. 9, 3469 (2018). 10.1038/s41467-018-05927-6 30150695 PMC6110746

[18] L. Kurzawa, B. Vianay, F. Senger, T. Vignaud, L. Blanchoin, and M. Théry, Mol. Biol. Cell 28, 1825 (2017). 10.1091/mbc.e16-09-0672 28684608 PMC5526557

[19] B. Tajvidi Safa, C. Huang, A. Kabla, and R. Yang, R. Soc. Open Sci. 11, 231074 (2024). 10.1098/rsos.231074 38660600 PMC11040246

[20] M. McCord and J. Notbohm, Soft Matter 22, 458 (2026). 10.1039/D5SM01139F 41392955 PMC12771545

[21] I. Haase, R. Evans, R. Pofahl, and F. M. Watt, J. Cell Sci. 116, 3227 (2003). 10.1242/jcs.00610 12829742

[22] T. J. Stefonek-Puccinelli and K. S. Masters, in *2009 Annual International Conference of the IEEE Engineering in Medicine and Biology Society* (IEEE, 2009), pp. 1167–1171. 10.1109/IEMBS.2009.5332607 19963489

[23] L. E. Wickert, S. Pomerenke, I. Mitchell, K. S. Masters, and P. K. Kreeger, Sci. Rep. 6, 20139 (2016). 10.1038/srep20139 26832302 PMC4735862

[24] C. S. Kim, I. P. Mitchell, A. W. Desotell, P. K. Kreeger, and K. S. Masters, FASEB J. 30, 2580 (2016). 10.1096/fj.201600252 27025961 PMC4904288

[25] C. S. Kim, X. Yang, S. Jacobsen, K. S. Masters, and P. K. Kreeger, Bioeng. Transl. Med. 4, e10138 (2019). 10.1002/btm2.10138 31572796 PMC6764804

[26] E. Lång, A. Połeć, A. Lång, M. Valk, P. Blicher, A. D. Rowe, K. A. Tønseth, C. J. Jackson, T. P. Utheim, L. M. Janssen *et al.*, Nat. Commun. 9, 3665 (2018). 10.1038/s41467-018-05578-7 30202009 PMC6131553

[27] R. Kobayashi, E. Hoshikawa, T. Saito, O. Suebsamarn, E. Naito, A. Suzuki, S. Ishihara, H. Haga, K. Tomihara, and K. Izumi, FEBS Open Bio 13, 1469 (2023). 10.1002/2211-5463.13653 PMC10392064 37243482

[28] S. L. Jeppe Knudsen, A. S. Wai Mac, L. Henriksen, B. v Deurs, and L. M. Grøvdal, Growth Factors 32, 155 (2014). 10.3109/08977194.2014.952410 25257250

[29] K. Vazquez, A. Saraswathibhatla, and J. Notbohm, Sci. Rep. 12, 2474 (2022). 10.1038/s41598-022-06504-0 35169196 PMC8847350

[30] C. Blanch-Mercader, R. Vincent, E. Bazellières, X. Serra-Picamal, X. Trepat, and J. Casademunt, Soft Matter 13, 1235 (2017). 10.1039/C6SM02188C 28098306

[31] L. Rossetti, S. Grosser, J. F. Abenza, L. Valon, P. Roca-Cusachs, R. Alert, and X. Trepat, Nat. Phys. 20, 1659 (2024). 10.1038/s41567-024-02600-2

[32] X. Kong, Z. Zhong, and C. Fang, Biophys. J. 124, 1995 (2025). 10.1016/j.bpj.2025.04.030 40319351 PMC12256876

[33] T.-K. N. Phung, J. A. Mitchel, M. J. O'Sullivan, and J.-A. Park, Biol. Open 12, bio059727 (2023). 10.1242/bio.059727 37014330 PMC10151827

[34] W. Xi, S. Sonam, T. Beng Saw, B. Ladoux, and C. Teck Lim, Nat. Commun. 8, 1517 (2017). 10.1038/s41467-017-01390-x 29142242 PMC5688140

[35] P. Pandya, J. L. Orgaz, and V. Sanz-Moreno, Curr. Opin. Cell Biol. 48, 87 (2017). 10.1016/j.ceb.2017.06.006 28715714 PMC6137077

[36] C. Santa-Cruz Mateos, A. Valencia-Exposito, I. M. Palacios, and M. D. Martin-Bermudo, PLoS Genet. 16, e1008717 (2020). 10.1371/journal.pgen.1008717 32479493 PMC7263567

[37] D. W. Dumbauld, T. T. Lee, A. Singh, J. Scrimgeour, C. A. Gersbach, E. A. Zamir, J. Fu, C. S. Chen, J. E. Curtis, S. W. Craig *et al.*, Proc. Natl. Acad. Sci. U. S. A. 110, 9788 (2013). 10.1073/pnas.1216209110 23716647 PMC3683711

[38] C. Grashoff, B. D. Hoffman, M. D. Brenner, R. Zhou, M. Parsons, M. T. Yang, M. A. McLean, S. G. Sligar, C. S. Chen, T. Ha *et al.*, Nature 466, 263 (2010). 10.1038/nature09198 20613844 PMC2901888

[39] K. E. Rothenberg, D. W. Scott, N. Christoforou, and B. D. Hoffman, Biophys. J. 114, 1680 (2018). 10.1016/j.bpj.2018.02.019 29642037 PMC5954296

[40] G. Kadamur and E. M. Ross, Annu. Rev. Physiol. 75, 127 (2013). 10.1146/annurev-physiol-030212-183750 23140367

[41] F. Hong, B. D. Haldeman, D. Jackson, M. Carter, J. E. Baker, and C. R. Cremo, Arch. Biochem. Biophys. 510, 135 (2011). 10.1016/j.abb.2011.04.018 21565153 PMC3382066

[42] W. H. Ziegler, U. Tigges, A. Zieseniss, and B. M. Jockusch, J. Biol. Chem. 277, 7396 (2002). 10.1074/jbc.M110008200 11741957

[43] L. Buonaguro, HEPAVAC Consortium *et al.*, Cytokine Growth Factor Rev. 36, 125 (2017). 10.1016/j.cytogfr.2017.06.010 28688773

[44] M. Poujade, E. Grasland-Mongrain, A. Hertzog, J. Jouanneau, P. Chavrier, B. Ladoux, A. Buguin, and P. Silberzan, Proc. Natl. Acad. Sci. U. S. A. 104, 15988 (2007). 10.1073/pnas.0705062104 17905871 PMC2042149

[45] M. Reffay, M.-C. Parrini, O. Cochet-Escartin, B. Ladoux, A. Buguin, S. Coscoy, F. Amblard, J. Camonis, and P. Silberzan, Nat. Cell Biol. 16, 217 (2014). 10.1038/ncb2917 24561621

[46] A. Saraswathibhatla, J. Zhang, and J. Notbohm, Phys. Rev. E 105, 024404 (2022). 10.1103/PhysRevE.105.024404 35291100

[47] T. B. Saw, A. Doostmohammadi, V. Nier, L. Kocgozlu, S. Thampi, Y. Toyama, P. Marcq, C. T. Lim, J. M. Yeomans, and B. Ladoux, Nature 544, 212 (2017). 10.1038/nature21718 28406198 PMC5439518

[48] J. Zhang, N. Yang, P. K. Kreeger, and J. Notbohm, APL Bioeng. 5, 036103 (2021). 10.1063/5.0047523 34396026 PMC8337086

[49] M. R. Nejad, L. J. Ruske, M. McCord, J. Zhang, G. Zhang, J. Notbohm, and J. M. Yeomans, Nat. Commun. 15, 3628 (2024). 10.1038/s41467-024-47702-w 38684651 PMC11059169

[50] J. R. Tse and A. J. Engler, Curr. Protoc. Cell Biol. 47, 10 (2010). 10.1002/0471143030.cb1016s47 20521229

[51] M. McCord, A. H. Khankhel, K. Kafkis, G. Radtke, S. Candan, H. A. Kumar, P. Derksen, M. Tam, K. Zhou, M. W. Merk, C. Franck, S. J. Streichan, and J. Notbohm, PLoS One 21, e0344657 (2026). 10.1371/journal.pone.0344657 41849440 PMC12998803

[52] J. Notbohm and M. McCord, *Protocol for Micropatterning of Cell Monolayers into Islands of Desired Size and Shape* (Protocols.io, 2026). 10.17504/protocols.io.eq2ly4nmwlx9/v3

[53] F. M. Pramotton, F. Robotti, C. Giampietro, T. Lendenmann, D. Poulikakos, and A. Ferrari, ACS Biomater. Sci. Eng. 5, 3922 (2019). 10.1021/acsbiomaterials.8b01346 33438431

[54] J. C. Crocker and D. G. Grier, J. Colloid Interface Sci. 179, 298 (1996). 10.1006/jcis.1996.0217

[55] E. Bar-Kochba, J. Toyjanova, E. Andrews, K.-S. Kim, and C. Franck, Exp. Mech. 55, 261 (2015). 10.1007/s11340-014-9874-2

[56] A. Saraswathibhatla, E. E. Galles, and J. Notbohm, Sci. Data 7, 197 (2020). 10.1038/s41597-020-0540-5 32581285 PMC7314837

[57] J. P. Butler, I. M. Tolic-Nørrelykke, B. Fabry, and J. J. Fredberg, Am. J. Physiol. 282, C595 (2002). 10.1152/ajpcell.00270.2001 11832345

[58] J. C. Del Alamo, R. Meili, B. Alonso-Latorre, J. Rodríguez-Rodríguez, A. Aliseda, R. A. Firtel, and J. C. Lasheras, Proc. Natl. Acad. Sci. U. S. A. 104, 13343 (2007). 10.1073/pnas.0705815104 17684097 PMC1940228

[59] J. Schindelin, I. Arganda-Carreras, E. Frise, V. Kaynig, M. Longair, T. Pietzsch, S. Preibisch, C. Rueden, S. Saalfeld, B. Schmid, J.-Y. Tinevez, D. J. White, V. Hartenstein, K. Eliceiri, P. Tomancak, and A. Cardona, Nat. Methods 9, 676 (2012). 10.1038/nmeth.2019 22743772 PMC3855844

[60] C. A. Schneider, W. S. Rasband, and K. W. Eliceiri, Nat. Methods 9, 671 (2012). 10.1038/nmeth.2089 22930834 PMC5554542

[61] Z. Püspöki, M. Storath, D. Sage, and M. Unser, *Focus on Bio-image Informatics* (Springer, Cham, Switzerland, 2016), p. 69. 10.1007/978-3-319-28549-8_3

[62] K. Burton, J. H. Park, and D. L. Taylor, Mol. Biol. Cell 10, 3745 (1999). 10.1091/mbc.10.11.3745 10564269 PMC25676

[63] S. Munevar, Y.-L. Wang, and M. Dembo, Mol. Biol. Cell 12, 3947 (2001). 10.1091/mbc.12.12.3947 11739792 PMC60767

